# A TNF-Regulated Recombinatorial Macrophage Immune Receptor Implicated in Granuloma Formation in Tuberculosis

**DOI:** 10.1371/journal.ppat.1002375

**Published:** 2011-11-17

**Authors:** Alexander W. Beham, Kerstin Puellmann, Rebecca Laird, Tina Fuchs, Roswita Streich, Caroline Breysach, Dirk Raddatz, Septimia Oniga, Teresa Peccerella, Peter Findeisen, Julia Kzhyshkowska, Alexei Gratchev, Stefan Schweyer, Bernadette Saunders, Johannes T. Wessels, Wiebke Möbius, Joseph Keane, Heinz Becker, Arnold Ganser, Michael Neumaier, Wolfgang E. Kaminski

**Affiliations:** 1 Department of Surgery, University of Göttingen, Göttingen, Germany; 2 Department of Hematology, Hemostasis, Oncology and Stem Cell Transplantation, Hannover Medical School (MHH), Hannover, Germany; 3 Institute for Clinical Chemistry, University of Heidelberg Medical Faculty Mannheim, Mannheim, Germany; 4 Department of Medicine, University of Göttingen, Göttingen, Germany; 5 Department of Dermatology, University of Heidelberg Medical Faculty Mannheim, Mannheim, Germany; 6 Institute of General Pathology and Pathophysiology, Russian Academy of Medical Sciences, Moscow, Russia; 7 Department of Pathology, University of Göttingen, Göttingen, Germany; 8 Medicine, Central Clinical School, Centenary Institute of Cancer Medicine and Cell Biology, Sydney, Australia; 9 Department of Nephrology/Rheumatology, University of Göttingen, Göttingen, Germany; 10 Max-Planck-Institute of Experimental Medicine, Department of Neurogenetics, Göttingen, Germany; 11 Trinity College Dublin, Institute of Molecular Medicine, College Green, Dublin, Ireland; Portland VA Medical Center / Oregon Health and Science University, United States of America

## Abstract

Macrophages play a central role in host defense against mycobacterial infection and anti- TNF therapy is associated with granuloma disorganization and reactivation of tuberculosis in humans. Here, we provide evidence for the presence of a T cell receptor (TCR) αβ based recombinatorial immune receptor in subpopulations of human and mouse monocytes and macrophages. *In vitro*, we find that the macrophage-TCRαβ induces the release of CCL2 and modulates phagocytosis. TNF blockade suppresses macrophage-TCRαβ expression. Infection of macrophages from healthy individuals with mycobacteria triggers formation of clusters that express restricted TCR Vβ repertoires. *In vivo*, TCRαβ bearing macrophages abundantly accumulate at the inner host-pathogen contact zone of caseous granulomas from patients with lung tuberculosis. In chimeric mouse models, deletion of the variable macrophage-TCRαβ or TNF is associated with structurally compromised granulomas of pulmonary tuberculosis even in the presence of intact T cells. These results uncover a TNF-regulated recombinatorial immune receptor in monocytes/macrophages and demonstrate its implication in granuloma formation in tuberculosis.

## Author Summary

Infection with mycobacteria results in a host response which results in the formation of granulomas, highly organized structures characterized by the presence of macrophages, which are considered to rely solely on invariant immune receptors. On the other hand, the presence of variable immune receptors is required for granuloma formation but this process is not solely dependent on T cells. Furthermore, TNF is required for the maintenance of the mycobacterial granuloma structure in humans. We now find evidence for subpopulations of human and mouse macrophages that express variable αβ T cell receptors (TCRαβ). Engagement of the macrophage-TCRαβ triggers CCL2 release and phagocytosis of baits directed to this receptor is enhanced. TCRαβ bearing macrophages accumulate in human tuberculosis granulomas and anti-TNF treatment of macrophages results in downregulation of the TCRαβ, which is associated with caspase 3 cleavage and suppression of TCRξ. Anti-TNF treatment reduces mycobacteria induced cluster formation of TCRαβ positive macrophages, which is in line with reduced granuloma formation in rag1^–/–^(T cell rag1^+/+^) and TNF^–/–^(T cell TNF^+/+^) chimeric mice. Consequently, both chimeras show reduced CCL2 staining after mycobacterial infection. In summary, we have identified a recombinatorial immunoreceptor in monocytes/macrophages and demonstrate its implication in mycobacterial infection.

## Introduction

Macrophages are key players in major chronic inflammatory diseases including tuberculosis, atherosclerosis and rheumatoid arthritis. Based on their myeloid origin and professional phagocytic activity they are traditionally regarded as a pillar of innate immunity [1]. Tuberculosis is an infectious disease that in 2008 afflicted more than nine million individuals worldwide and claimed the lives of an estimated 1.3 million patients [2]. The disease is caused by mycobacteria that are efficiently contained by macrophages in highly organized immune structures, the tuberculous granulomas. Ample evidence indicates that the generation and maintenance of tuberculous granulomas require TNF [3], [4]. Moreover, reactivation of the disease by therapeutic TNF blockade is associated with disruption of the granuloma architecture that ultimately leads to spreading of the mycobacteria into the surrounding tissue [5].

Within the tuberculous granuloma, cellular immunity to mycobacteria is thought to be solely under the direction of T cells which orchestrate the macrophage host response to the pathogen. However, selective T cell depletion and reconstitution experiments in murine models of tuberculosis point to the involvement of variable host defense mechanisms in the control of mycobacterial infection beyond T cells [6]-[8]. The recent demonstration by our laboratory and others that neutrophils and eosinophils express T cell receptors (TCR) which are generated by V(D)J recombination has provided evidence for the existence of variable immune receptors outside lymphocytes [9]-[11].

These findings and the possibility that variable immune defense mechanisms outside T cells are implicated in the development of the tuberculous granuloma raise the question as to whether macrophages possess a molecular machinery for variable host defense. Here, we report that subpopulations of monocytes and macrophages express a recombinatorial TCRαβ which is TNF regulated and demonstrate a role of this novel immune receptor in the macrophage host response to mycobacteria and the formation of the tuberculous granuloma.

## Results

### Subpopulations of monocytes in the circulation and macrophages express the TCRαβ

**Figure 1:**
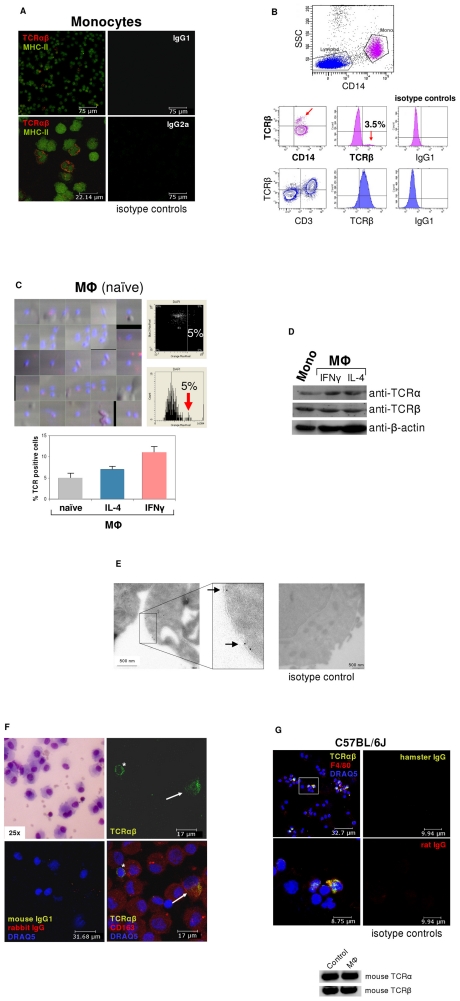
ΤCRαβ expression by subpopulations of human and murine monocytes/macrophages. (**A**) Fluorescence immunocytochemistry demonstrating that a ∼5% subpopulation of human peripheral blood CD14^+^/MHC-II^+^ monocytes expresses the ΤCRαβ. CD14-MACS purified peripheral blood monocytes were isolated from a healthy donor and double-immunostained with Abs against the ΤCRαβ (red) and MHC-II (green). Isotype controls for the anti-ΤCRαβ and anti-MHC-II antibodies are shown (right). Scale bars are indicated. Data shown are representative of n = 12 donors. (**B**) Flow cytometry of peripheral blood mononuclear cells from a healthy individual demonstrates the presence of the TCRβ on the surface of a CD14^+^ monocyte subpopulation (3.5%, red arrows). Staining for TCRβ and lineage surface markers are shown. CD14^+^ monocytes are in pink color, CD3^+^ lymphocytes in blue. (**C**) (Left) Laser scanning cytometry (LSC) of unstimulated (naïve) monocyte-derived macrophages stained for ΤCRαβ (red). Nuclei are counterstained with DAPI (blue). The cytometric analysis shows a subpopulation (5%) with high fluoresence indicative of ΤCRαβ positive naïve macrophages (top right) which is highlighted in the histogram below (arrow). (Bottom) LSC of naïve and IL-4 (10 ng/ml) or IFNγ (1000 U/ml) stimulated monocyte-derived macrophages, respectively, cultured for 6 days. The percentage of ΤCRαβ^+^ cells in each macrophage population is shown for three healthy individuals. (**D**) Detection of the TCR α- and β-chain in CD14^+^ monocytes and IFNγ or IL-4 polarized macrophages by immunoblot. β-actin, loading control. (**E**) Immunogold electron microscopy demonstrating the presence of the TCR α-subunit on the cell surface of a human IFNγ stimulated macrophage (arrows). (Right) isotype control. (**F**) Immunocytochemical double-staining reveals the presence of the TCRαβ (green) in alveolar macrophages from a 45 year old male with normal BAL cytology. Shown is a ΤCRαβ^+^ alveolar macrophage (top right, arrow) next to a ΤCRαβ^+^ T cell (asterisk). The merged image (bottom right) demonstrates that the majority of the cells express the macrophage marker CD163 (red). Giemsa-staining of the BAL cytospin preparation and isotype controls are shown in the left panel. The results are representative of three individuals. Nuclei (blue), DRAQ5. (**G**) Confocal immunofluorescence microscopy shows ΤCRαβ expression in murine macrophages. Spleen macrophages pooled from three normal C57BL/6 J mice were CD11b-MACS purified and immunofluorescence double-staining was performed using the anti-macrophage antibody F4/80 (red) and an anti-mouse TCRβ antibody that recognizes a common epitope of the murine TCRαβ complex (green). The outlined area is shown at a higher magnification. Nuclei (blue) are counterstained with DRAQ5. Isotype controls are shown. (Bottom) RT-PCR demonstrating expression of the murine TCRα and TCRβ constant chain genes in CD11b-MACS purified spleen macrophages (MΦ) from C57Bl6/J mice. Ly6G^+^ neutrophils are shown as positive control.

To assess the possibility that monocytes, like granulocytes [9]-[11], express the TCRαβ, we isolated human CD14^+^ monocytes from healthy donors (n = 12). Expression of the TCRαβ in peripheral blood monocytes was assessed in MACS-CD14^+^ purified cells by immunocytochemistry using antibodies to TCRα/TCRβ and MHC-II. Utilizing this approach, we consistently detected a ∼5% cell fraction that displayed bright TCRαβ^+^ expression in freshly isolated CD14^+^ monocytes which showed co-expression of MHC-II (Figure 1A). Purity of isolated CD14^+^ monocytes was routinely >99.5% as determined by flow cytometry (Figure S1A). We next characterized the TCRαβ expressing monocyte subpopulation in PBMC by flow cytometry. Consistent with immunocytochemistry, CD14^+^ cells from three normal subjects displayed positive staining for TCRβ in a 3–4% subfraction which did not exhibit staining for the T cell marker CD3 (Figure 1B, Figure S1B). We then determined expression of the ΤCRαβ in monocyte-derived macrophages from three healthy donors. For this, monocytes were differentiated into naïve, IFNγ activated and IL-4 activated macrophages, respectively, on glass slides for a period of 6 days [12] and stained for TCRαβ. To quantify the TCRαβ^+^ populations in adherent macrophages, laser scanning cytometry (LSC) was performed on the slides that were immunostained with Alexa 555-labeled secondary antibodies. LSC analysis demonstrated that a 5% subpopulation of naïve macrophages exhibited high fluorescence indicative of TCRαβ expression (Figure 1C, Figure S1C). In contrast, the fraction of TCRαβ bearing macrophages was significantly higher in the IL-4 (9%) and IFNγ activated macrophages (11%). Thus, activation of macrophages induces an increase in the subpopulation of TCRαβ expressing macrophages.

Immunoblot analysis confirmed the expression of the TCR α- and β-chains in monocytes and macrophages (Figures 1D). In line with this, immunogold electron microscopy using an antibody to the TCR α-subunit revealed specific staining in monocyte-derived macrophages (Figure 1E), which was detectable on the cell surface. Next we looked for evidence of TCRαβ expressing macrophages *in vivo*. For this, we performed double-immunostaining for the macrophage marker CD163 and the TCRαβ in bronchoalveolar lavage (BAL) fluid obtained from three individuals with normal BAL cytology. In fact, we found that a 5–15% subpopulation of alveolar macrophages showed positive staining for the TCRαβ (Figure 1F, Figures S1D and E) demonstrating that tissue macrophages are capable of expressing the TCR under physiological conditions.

We then tested whether the TCR is also expressed in macrophages from the mouse. Consistent with our findings in humans, immunocytochemistry demonstrated the presence of the TCRαβ in a 5–10% subfraction of spleen macrophages from C57BL/6J mice (n = 3) and RT-PCR revealed mRNA expression of both the TCR α- and β-constant chains in splenic macrophages (Figure 1G).

Together, these results reveal that subpopulations of peripheral blood monocytes and *in vitro* activated monocyte-derived macrophages constitutively express the TCRαβ. Moreover, they demonstrate the presence of ΤCRαβ bearing macrophages in normal human tissue, as exemplified for the lung, and provide evidence for TCRαβ expression by murine macrophages.

### Monocytes/macrophages express variable TCR α- and β-repertoires

**Figure 2:**
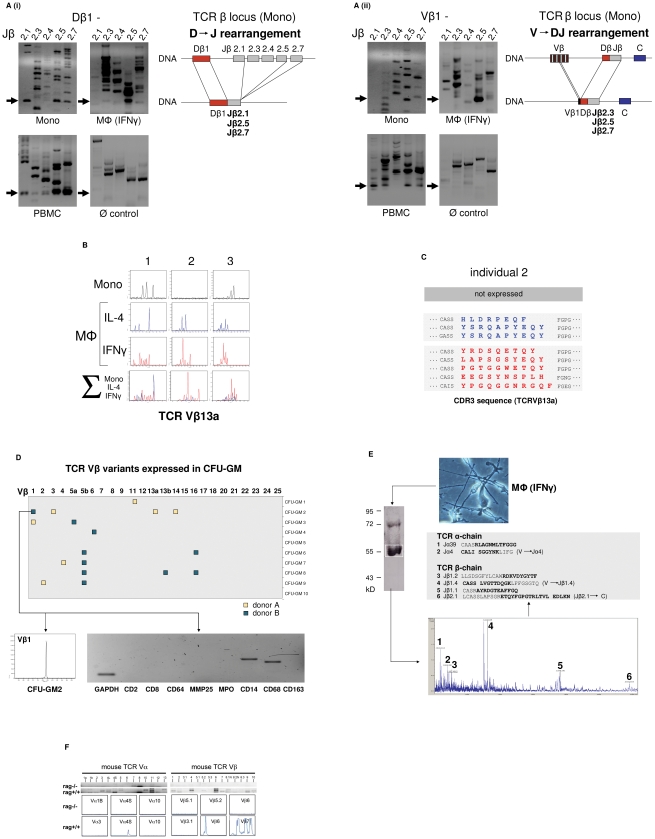
The monocyte/macrophage TCRαβ is a recombinatorial receptor. (**A**) Detection of D → J (i) and V → DJ (ii) rearrangements in the TCRβ gene locus of human CD14^+^ monocytes and IFNγ macrophages. Arrows denote the presence of Dβ1→ Jβ and Vβ1→ Jβ rearrangements which were confirmed by sequencing. Genomic organization of the identified rearrangements is schematically drawn. Peripheral blood mononuclear cells (PBMC), positive control. HepG2 cells, Ø control. (**B**) Expression of individual-specific TCR Vβ repertoires by monocytes, IL-4 macrophages (blue) and IFNγ macrophages (red) from three healthy donors (1–3) representatively shown for Vβ13a. A scaled synopsis of the three cell populations is shown at the bottom (∑). (**C**) TCR clonotype analysis by sequencing of the antigen-binding CDR3 loop of Vβ13a representatively shown for individual 2. IL-4 (blue) and IFNγ activated macrophages (red) express completely different Vβ13a clonotypes. The Vβ13a chain is not expressed by the monocytes of this individual (cf. B). Colored letters represent deduced amino acid sequences of the newly identified CDR3_β_ regions (GenBank Acc. No. JF923737-JF923744). **D**) Expression of rearranged TCR Vβ CDR3 clonotypes in granulocyte/macrophage progenitor colonies (CFU-GM) obtained from CD34^+^ progenitors of two healthy individuals (A and B). Filled boxes indicate positive expression of at least one of the 25 known human TCR Vβ chains (x-axis) in a single colony. Colonies are identified by numbering on the y-axis. The repertoires for each of the expressed Vβ chains were determined by length variant analysis of the antigen-binding CDR3_β_ region. The detailed Vβ repertoire is representatively shown for colony CFU-GM2 (donor B). The repertoires of additional CFU-GM colonies are summarized in Figure S2B. RT-PCR lineage marker expression profiling documents the monocytic nature of this granulocyte/macrophage progenitor colony. CD2, CD8: T lymphoid markers; MMP25, MPO: granulocyte markers; CD14, CD68, CD163: monocyte markers. (**E**) Direct mass spectrometric identification of multiple TCR Vα- and Vβ-chain variants in human macrophages. Protein lysates from IFNγ macrophages of a healthy donor were immunoprecipitated using an anti-TCRβ antibody and the predicted 58 kD band (boxed) was analyzed by MALDI-TOF mass spectrometry. Peaks 1–6 represent TCR Vα- and Vβ-specific peptide fragments whose amino acid sequence identities with known TCR Vαβ-clonotypes are bolded. In three cases (2, 4 and 6), the identified peptides span V→ J and J→ C junctions (denoted by a gap) indicative of genomic rearrangements in the macrophage TCRα and -β loci. (**F**) Peritoneal macrophages from C57Bl6/J mice (rag1^+/+^) but not recombination defective rag1^–/–^ mice express Vα (left) and Vβ repertoires (right) as evidenced by TCR V-chain mRNA expression profiling (top) and CDR3 spectratyping of representative TCR Vα- and Vβ-chains (bottom). Peritoneal macrophages were pooled from three rag1^+/+^ mice and an equal number of rag1^–/–^ mice, respectively.

We next investigated whether the TCRαβ expressed by monocytes/macrophages represents a variable receptor. For this, we tested whether CD14^+^ monocytes and IFNγ macrophages have a rearranged TCRβ locus. Using a PCR assay based on the protocols established by van Dongen et al. [13] in combination with sequencing of specific amplification products, evidence for TCRβ locus genomic Dβ1→Jβ (Figure 2A i) and Vβ1→ Jβ (Figure 2A ii) recombination was found in both the monocyte and IFNγ macrophage fractions. Length spectratyping of the antigen-binding complementary determining region 3 (CDR3) is a well-established method for the assessment of TCR repertoire diversity in defined variable chains [14]. Representative Vβ13a CDR3 spectratype analysis in IL-4 or IFNγ primed macrophages from healthy individuals (n = 3) revealed monoclonal and oligoclonal repertoires and varied in the same donor depending on IL-4 or IFNγ activation (Figure 2B). Sequencing of the expressed Vβ13a CDR3_β_ clonotypes in one subject (GenBank Acc. No. JF923737-JF923744) indeed revealed marked differences between IL-4 and IFNγ polarized macrophages (Figure 2C). Of note, quantitation of the expressed length variants, respectively, in all three individuals consistently demonstrated increased repertoire TCR Vα and Vβ diversity in IFNγ activated macrophages relative to monocytes and IL-4 macrophages (Figure S2A).

Evidence for TCRβ locus rearrangement in mature CD14^+^ monocytes and our previous observation of a rearranged TCRαβ in neutrophils [9] strongly suggested that TCR recombination occurs already at an early stage of myeloid development. To test this possibility, burst-forming unit-erythroid (BFU-E) and granulocyte/macrophage progenitor colonies (CFU-GM) were generated from CD34^+^ hematopoietic progenitor cells of two normal donors in two independent experiments. TCR Vβ mRNA expresssion profiling was performed on 10 randomly selected colonies from each individual. CDR3_β_ length spectratyping revealed expression of single or few rearranged Vβ clonotypes in 50% (donor A) and 70% (donor B), respectively, of the CFU-GM analyzed (Figure 2D, Figure S2B). No TCR gene expression was observed in any of the BFU-E colonies tested (data not shown). The majority of the CFU-GM displayed a monoclonal expression pattern consistent with the clonogenic nature of the myeloblasts and monoblasts in this assay. This indicates that TCRβ locus rearrangement and expression of individual-specific Vβ repertoires occurs already during the early phase of *in vitro* myeloid lineage differentiation.

We next sought evidence for TCR α- and β-chain variability at the protein level. For this, the TCRαβ from macrophages of healthy donors was immunoprecipitated and subjected to MALDI-TOF mass spectrometry. Using this proteome profiling approach, we identified two peptides that showed partial sequence identity with known variable TCR α-chain fragments (Jα4, Jα39) and a total of four distinct peptides displaying partial sequence identity with variable TCR β-chain fragments (Jβ1.1, Jβ1.2, Jβ1.4, Jβ2.1) (Figure 2E). Three of the peptides (Jα4, Jβ1.4, Jβ2.1) spanned V→ J and J→ C junctions indicating that they originated from rearranged TCR α and β loci. These TCRαβ proteome profiling results are consistent with the presence of multiple TCR α- and β-chain variants in human macrophages.

In mice, TCR Vαβ mRNA profiling and CDR3 spectratyping of purified peritoneal macrophages confirmed the presence of diverse Vα and Vβ repertoires in wildtype macrophages. In contrast, no evidence for TCR Vαβ repertoire expression was found in macrophages from rag1^–/–^ mice which are incapable of rearranging their immune receptor loci and thus lack a variable TCRαβ (Figure 2F).

In summary, the combined results from TCRβ VDJ rearrangement analyses, Vαβ CDR3 mRNA expression profiling and mass spectrometric TCRαβ peptide profiling indicate that the TCRαβ identified in human and murine macrophages is expressed as a variable receptor.

### Macrophage-TCRαβ engagement induces CCL2 release

**Figure 3:**
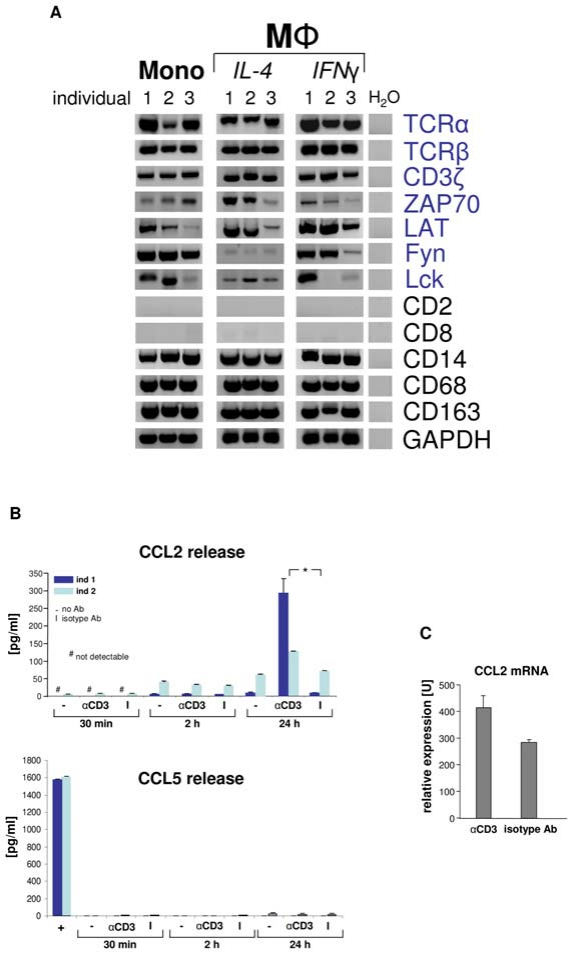
Engagement of the macrophage TCRαβ induces CCL2 release. (A) Circulating human monocytes (Mono), IL-4 activated and IFNγ activated macrophages (MΦ), respectively, constitutively express the genes for the TCRαβ chains and integral components of the TCR signalling complex (CD3ζ, ZAP70, LAT, Fyn, Lck). RT-PCR profiling is shown for three representative healthy individuals (1–3). Expression of T cell (CD2, CD8) and monocyte/macrophage marker genes (CD14, CD68, CD163) are demonstrated as reference. Peripheral blood monocytes were isolated by CD14-MACS and differentiation into Th1 (IFNγ) and Th2 (IL-4) polarized MΦ was induced for 6 days. ZAP70, CD3ζ associated protein kinase 70; LAT, linker for T cell activation; Fyn, Lck, *src* family tyrosine kinases; H_2_O, negative control. (**B**) CD3 mediated TCR activation induces selective CCL2 release from macrophages. Aliquots of 5×10^5^ IFNγ macrophages were incubated with soluble antibodies to CD3, isotype control antibodies (I) or in the absence of antibodies (-) for the indicated timepoints. CCL2 and 14 additional cytokines (Table S1) were determined in the supernatant by multiplex cytokine assay. The near absence of the T cell secretory protein CCL5 documents that macrophages were virtually free of T lymphocytes (bottom). Macrophages were collected from two healthy donors (ind 1, ind 2). +, CD3^+^ T cells (positive control). *p<0.05. (**C**) TCR engagement upregulates CCL2 gene expression in macrophages as assessed by quantitative RT-PCR (qPCR). The results shown represent the qPCR analysis of IFNγ macrophages from three healthy donors that were stimulated with anti-CD3 antibodies for 24 h.

Given the presence of the TCRαβ ligand binding subunits in monocytes/macrophages, we tested whether these cells also express constituents of the TCR signaling pathway. RT-PCR expression profiling revealed expression of all critical components of the TCR signal transduction machinery including CD3ζ, ZAP70, LAT, Fyn, and Lck, respectively, in monocytes and macrophages from three randomly selected healthy donors (Figure 3A). To explore whether the TCRαβ complex is operative in macrophages, we next tested whether specific TCR engagement has an impact on the secretion of effector molecules by IFNγ macrophages. Analysis of the secretory pattern of a defined panel of cytokines, chemokines and growth factors (n = 15, Table S1, Figure S3) in response to canonical TCR stimulation with anti-CD3 antibodies revealed enhanced secretion of the major monocyte chemoattractant CCL2 (MCP-1) within 24 h (Figure 3B). No detectable effect was observed for any of the other studied macrophage effector proteins or the secretory protein CCL5, which served as a marker for potential T cell contamination, indicating that engagement of CD3 dependent macrophage-TCRαβ signaling triggers selective secretion of CCL2 (Table S1, Figure S3). Consistent with this, anti-CD3 antibodies induced CCL2 gene expression in IFNγ macrophages (Figure 3C). Thus, specific engagement of the TCRαβ in macrophages induces gene expression and secretion of the monocyte chemoattractant CCL2 indicating that the TCRαβ signal transduction pathway in macrophages is functional.

### The macrophage-TCRαβ modulates phagocytosis

**Figure 4:**
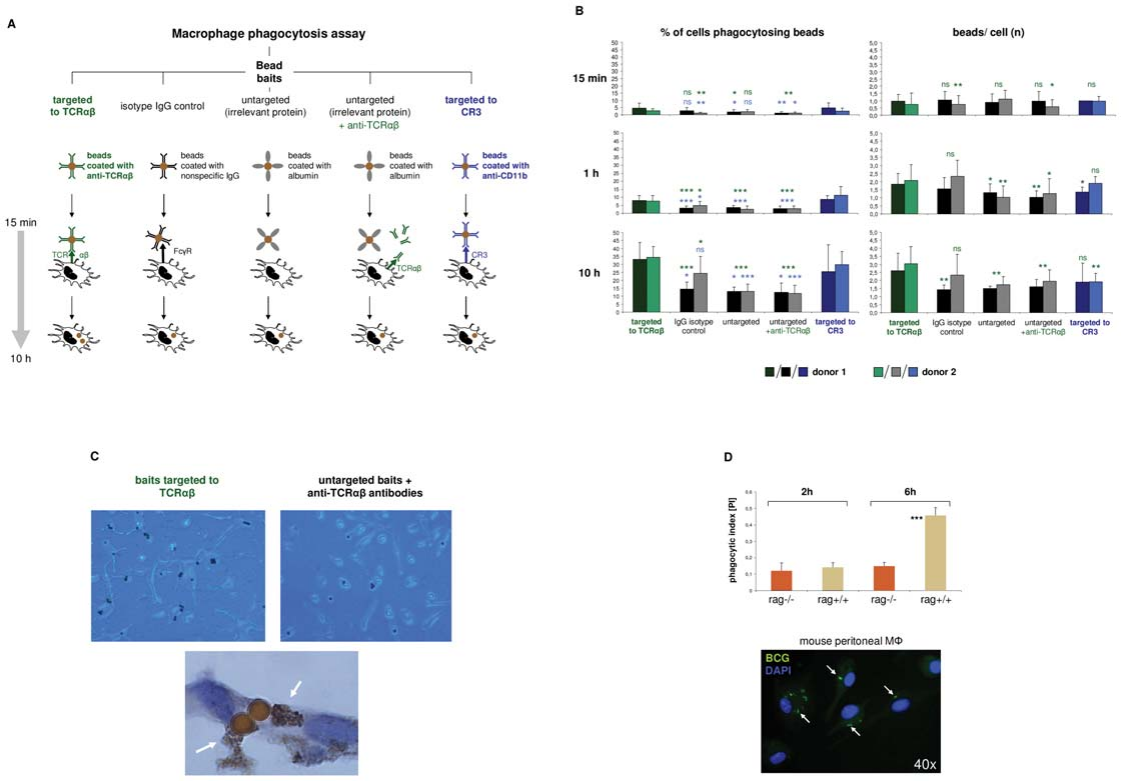
The macrophage TCRαβ modulates phagocytosis. (A) Schematic representation of the phagocytosis assay used for targeting of baits to the macrophage TCRαβ. IFNγ polarized macrophages were challenged with polystyrene bead baits (Ø 4.5 µm) coated with anti-TCRαβ antibodies for 15 min, 1 h and 10 h, respectively, and uptake of beads was recorded. Beads coated with nonspecific IgG antibodies, potentially binding to the Fcγ receptor (FcγR), anti-CD11b antibodies targeting the complement receptor 3 (CR3) and albumin (irrelevant protein) served as controls. In addition, macrophages were challenged with uncoupled anti-TCRαβ antibodies in the presence of albumin-coated bead baits. (**B**) Enhanced phagocytosis of baits targeted to the macrophage TCRαβ. Shown is the time course analysis of the percentage of phagocytosing cells (left) and the bead/cell ratios (right) in IFNγ macrophages that were challenged with bead baits as detailed in (A). IFNγ macrophages from two healthy donors were incubated with beads (MΦ:beads  =  1∶1, 5 µg Ab/10^7^ beads) for the indicated timepoints. Quantitation of phagocytosed beads was performed by bright field microscopy of at least 12 randomly selected fields of vision. P values refer to beads targeted to the TCR (green) or the CR3 (blue). *p<0.05, ** p<0.01, ***p<0.001; ns, not significant. (**C**) Representative unstained cytospin preparations (40x) of IFNγ macrophages from donor 1 that were challenged with bead baits targeted to the TCRαβ (top left) or albumin-coated beads in the presence of uncoupled anti- TCRαβ antibodies (control, top right). DAB immunostaining of two adjacent macrophages (bottom, 100x) demonstrates the presence of the TCRαβ (arrows) in immediate proximity to two ingested beads that were targeted to this immune receptor. The quantitative analysis of phagocytosed beads from this individual is shown in (B). (**D**) Reduced phagocytosis of *M. bovis BCG* by macrophages from recombination defective rag1^–/–^ mice which lack the recombinatorial TCRαβ. Thioglycollate-elicited peritoneal macrophages from rag1^–/–^ mice and rag1^+/+^ wildtype control mice were infected with FITC-labeled *M. bovis BCG* (MΦ:*BCG*  =  1∶10) and the phagocytic index was determined at the indicated timepoints by digital analysis of fluorescent images. ***p<0.001. The data are based on the quantitative analysis of 7 pooled rag1^–/–^ mice and an equal number of rag1^+/+^ mice. The fluorescent image (bottom) representatively shows rag1^+/+^ peritoneal macrophages after 6 h of infection with *BCG*. Arrows highlight ingested *BCG* mycobacteria. Nuclei, DAPI (blue).

Next, we investigated whether the TCRαβ interferes with the phagocytic activity of macrophages. For this, IFNγ activated macrophages from two healthy donors were challenged with standardized phagocytosis baits (polystyrene beads, Ø 4.5 µm) for 15 min, 1 h and 10 h, respectively. To induce physical interaction of the baits with the macrophage-TCRαβ, the beads were coated with anti-TCRα/anti-TCRβ antibodies (Figure 4A). Identical beads coupled to equal amounts of nonspecific IgG antibodies or albumin (irrelevant protein) were used as controls. In addition, macrophages were challenged with albumin-coated beads in the presence of anti-TCRαβ antibodies that were not physically coupled to baits. As a positive control, baits were targeted to the known mediator of phagocytosis complement receptor 3 (CR3) utilizing antibodies to its subunit CD11b. Using this approach, we observed in both donors that the number of phagocytosing macrophages was significantly increased after 1 h when baits were directed to the TCR (1.4–3.0 fold vs. controls) (Figure 4B). This increase was already detectable after 15 min, however, did not reach statistical significance for all controls. A similar augmentation of phagocytosis was seen when beads were targeted to the CR3 (1.2–4.5 fold vs. controls). In addition, we found that after 10 h baits directed to the TCR had elevated bead/cell ratios relative to controls (1.3–1.8 fold). These findings suggest that binding of baits to the TCRαβ facilitates phagocytosis. Consistent with this, we noted that phagocytosis was unaffected when anti-TCRαβ antibodies were not physically linked to beads (Figure 4B,C). As expected, we found evidence for close proximity of ingested baits to the TCRαβ (Figure 4C).

In light of the observation that the TCR facilitates phagocytosis, we next investigated whether and to which degreee complete ablation of the TCR has a negative effect on the macrophage phagocytic capacity. For this, peritoneal macrophages from recombination defective rag1^–/–^ mice (n = 7), which lack the TCRαβ (Figure 2F) were incubated with FITC-labeled *Mycobacterium bovis Bacille-Calmette-Guérin* (*BCG*). We used mycobacteria as baits because they represent classical macrophage pathogens and, like CCL2, are critically implicated in macrophage-driven granuloma formation [15]. Consistent with the findings in the bead targeting experiments phagocytosis of *M. bovis BCG* was significantly reduced in rag1^–/–^ macrophages compared to recombination-competent rag1^+/+^ control mice (n = 7) after 6 hours of infection (Figure 4D). Together, these bait targeting and ablation experiments strongly suggest roles of the TCRαβ in the regulation of macrophage phagocytic activity.

### *M. bovis BCG* **infection induces formation of macrophage clusters that express restricted TCR Vβ repertoires *in vitro***

**Figure 5:**
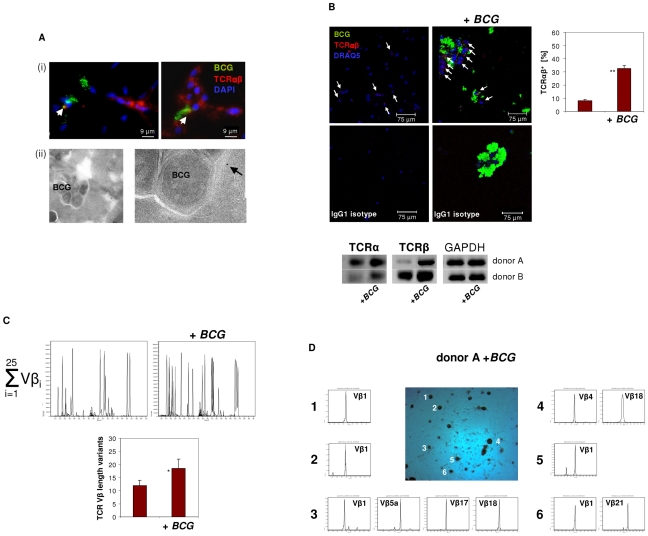
Infection of macrophages with *M. bovis BCG* induces TCRαβ expression *in vitro*. (**A**) Immunofluorescence double-staining demonstrating co-localization of *BCG* mycobacteria (green) and the macrophage-TCRαβ (red) after 6 days of infection (i). Nuclei are stained with DAPI. Arrows highlight two double-stained points of spatial proximity (yellow fluorescence). (ii) Immunogold electron microscopy of a *BCG* containing phagosome in an IFNγ macrophage. The presence of a single TCRαβ in the immediate neighborhood of a *BCG* mycobacterium is shown at a higher magnification (arrow). (**B**) Confocal image of a TCRαβ expressing macrophage cluster induced in response to i*n vitro* infection with *BCG*. Uninfected IFNγ macrophages from the same donor are shown left. White arrows highlight TCRαβ^+^ macrophages. A quantitative analysis of the percentage of TCRαβ^+^ cells (right) and isotype controls are shown. **p<0.01. IFNγ macrophages were incubated in the presence or absence of FITC-labeled *BCG* for 6 days. The results represent two healthy individuals (donors A and B). Donor B see Figure S4A. (Bottom) RT-PCR demonstrating increased expression of the TCR α- and β-chain genes in the *BCG* infected macrophages from both donors. GAPDH expression is shown as reference. (**C**) Synopsis of the TCR Vβ repertoires expressed by the *BCG* infected and uninfected macrophages shown in (B) assessed by CDR3 spectratyping (donor A). (Bottom) Quantitative analysis of all Vβ CDR3 length variants expressed by both donors . * p = 0.07. (**D**) TCR Vβ repertoire analysis of randomly selected *BCG*/macrophage clusters from donor A reveals expression of highly restricted TCR Vβ chain repertoires. *BCG*/macrophage clusters 1–6 were subjected to RT-PCR and CDR3 spectratyping. The identified TCR Vβ repertoires are shown for each individual cluster. Note that next to the Vβ1 only few additional Vβ chains are expressed. The single peaks are indicative of monoclonality. The results are representative of two healthy individuals (donors A and B). Donor B see Figure S4B.

Phagocytosis of mycobacteria by macrophages with subsequent granuloma formation are key features of host defense in tuberculosis infection. Given this and the above results suggesting implication of the macrophage-TCRαβ in phagocytosis, we next tested whether mycobacterial challenge has an impact on TCR expression in macrophages *in vitro.* We infected macrophages with FITC-labeled *M. bovis BCG* and noted that within 4–6 days macrophages routinely formed clusters (Figure 5). No clusters were formed in the absence of mycobacteria (Figure 5B). Bacilli frequently formed aggregates which may reflect fragmentation of *M. bovis BCG*. We found evidence for co-localization of *BCG* and the TCRαβ by immunocytochemistry (Figure 5A i) and immunogold electron microscopy (Figure 5A ii), however, this phenomenon was rare. Quantitative analysis of *BCG*-infected IFNγ macrophages from two healthy donors revealed a 4-fold increase of the percentage of TCRαβ^+^ macrophages relative to uninfected controls (8 vs. 32.5) (Figure 5B, Figure S4A). Consistent with this, we found increased expression of both the TCR α and the β constant chain genes in the macrophages that were infected with *BCG*. Moreover, CDR3 spectratyping of all 25 TCR Vβ chains showed a noticeable but not significant increase in the number of expressed Vβ CDR3 length variants (12 vs 18.5) in *BCG*-infected macrophages (Figure 5C). *Ex situ* Vβ clonotype analysis of randomly selected *BCG*/macrophage clusters from both donors revealed consistent expression of highly restricted TCR Vβ chain repertoires (Figure 5D, Figure S4B). In particular, we noted a bias toward the use of the Vβ1 chain which was expressed by 10 out of the 11 (91%) *BCG*/macrophage clusters analyzed. Collectively, these results demonstrate that *in vitro M. bovis BCG* infection induces formation of TCRαβ bearing macrophage clusters that express highly restricted Vβ repertoires.

### Abundant accumulation of TCRαβ bearing macrophages in human granulomas of tuberculosis

**Figure 6:**
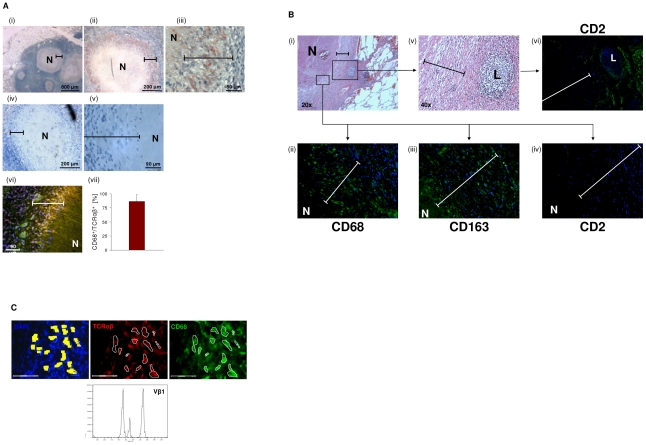
Massive accumulation of TCRαβ^+^ macrophages in the inner epithelioid cell corona of human tuberculous granulomas. (**A**) Presence of the macrophage-TCRαβ in circumscribed caseous tuberculous granulomas. Light microscopic images i-iii reveal intense red/brown immunostaining for the TCRαβ in the inner host-pathogen contact zone (bars) of the granulomas at various magnifications. (iv, v) Staining for CD2. N, necrotic caseous core. The lung sections were obtained from a patient with pulmonary tuberculosis and are representative of 10 out of 13 randomly selected patients (Table S2). Immunofluorescence double-staining for the TCRαβ (red) and the macrophage marker CD68 indicates that the TCRαβ^+^ cells are macrophages (vi, yellow, merged). (vii) Mean percentage of CD68^+^/TCRαβ^+^ cells in the inner epithelioid cell corona of circumscribed caseous tuberculous granulomas from 8 different patients. Percentages of double positive cells in this zone (CD68^+^/TCRαβ^+^ cells : total number of nuclei) were determined from three sections of each patient. (**B**) Single immunostaining for the markers CD68 and CD163 demonstrates that macrophages represent the vast majority of cells in the epithelioid cell corona of the caseous tuberculous granulomas (i–iii). Staining for CD2 reveals that T cells and natural killer cells are predominantly located in the outer segment of the corona (iv). A focal accumulation of lymphocytes (L) located in the peripheral corona zone is shown in the right box (i,v). Note positive staining for CD2 (vi). Bars in all images span the inner host-pathogen contact zone. N, necrotic core. Isotype controls are shown in Figure S5A. ii–iv,vi: 40x. (**C**) Laser microdissection of a 20–30 cell cluster of CD68^+^/TCRαβ^+^ double-immunostained macrophages (encircled) from the inner host-pathogen contact zone of a caseous tuberculous granuloma. TCRαβ^+^, red; CD68^+^, green; scale bar, 75 µm. Dissected cells are highlighted in yellow (left). *Ex situ* Vβ CDR3 spectratype analysis of the excised cells reveals expression of restricted TCR Vβ1 repertoires.

To examine whether the macrophage-TCR is implicated in host defense against mycobacteria *in vivo*, we next screened for the presence of TCRαβ expressing macrophages in tuberculous tissue. Lung sections of patients with pulmonary tuberculosis (n = 13, Table S2) were immunostained for TCRαβand the macrophage markers CD68 and CD163, respectively. Ten out of 13 patients showed abundant staining for TCRαβ in well-circumscribed caseous granulomas. Typically, the innermost segment of the epithelioid cell corona, which represents the front line of cellular defense against mycobacteria within tuberculous granulomas, exhibited intense TCRαβ^+^ staining (Figure 6A i-iii). Controls showed no staining for CD2 (Figure 6A iv,v). Quantitative analysis of TCRαβCD68 immunofluorescence double-staining revealed that on average 87% of the macrophages expressed the TCRαβ in this zone (Figures 6A vi, vii). Additional single immunofluorescence staining for CD68 and CD163 confirmed that the predominant cell type in the inner host-pathogen contact zone was macrophages (Figure 6B, Figure S5A). Consistent with this, T cells and NK cells were typically localized in the peripheral corona zone of caseous granulomas as assessed by CD2 immunostaining (Figure 6B). We found no evidence for TCRαβ expressing macrophages in a lymph node from an individual with reactivated *M. tuberculosis* infection triggered by anti-TNF therapy (adalimumab) (Figure S5B). In keeping with immunohistochemistry, *ex situ* clonotype analysis revealed expression of TCR Vβ mRNA repertoires in small clusters (20–30 cells) of immunostained CD68^+^ macrophages that were laser microdissected from the inner epithelioid cell zone (Figure 6C). Similarly as observed in *BCG* infected macrophage clusters *in vitro* (Figure 5D), we noted that the epithelioid zone macrophages predominantly express the TCR Vβ1 chain.

### TNF blockade suppresses macrophage-**TCR**αβ expression

**Figure 7:**
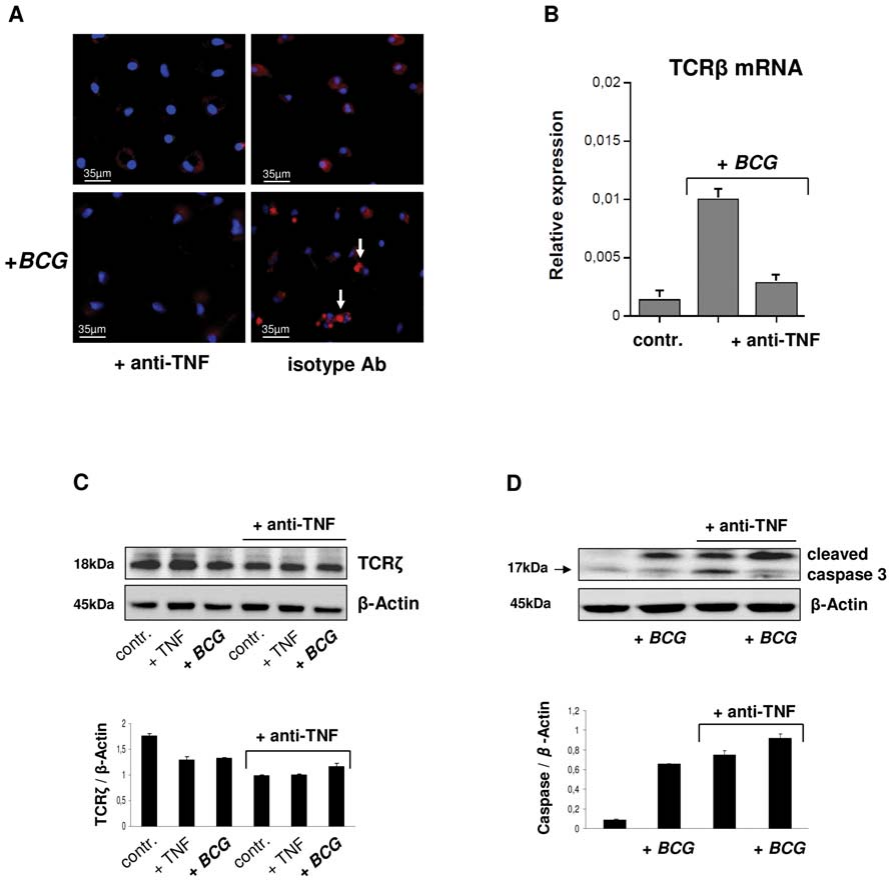
TNF blockade inhibits expression of the macrophage-TCRαβ. (**A**) Suppression of the TCRαβ in uninfected and *BCG* infected IFNγ macrophages in response to 2h treatment with the anti-TNF antibody infliximab (50 µg/ml). An irrelevant monospecific antibody (anti-CD20) was used as control. Macrophages were immunostained using an anti-TCRαβ antibody (Alexa 555-labeled, red). Representative fluorescent microscopy images are shown. Infection with *BCG* mycobacteria changes the staining pattern from diffuse surface staining to a bright central spot (arrows). Nuclei, DAPI (blue). (**B**) Anti-TNF treatment (infliximab) downregulates mRNA expression of the TCRβ constant chain in IFNγ macrophages as assessed by qPCR. Control, uninfected macrophages. (**C**) Downregulation of the ζ-subunit of the TCR signaling complex and increased levels of cleaved caspase 3 (**D**) in uninfected (controls, +TNF) and *BCG* infected IFNγ macrophages following TNF blockade (infliximab). Controls, untreated macrophages; +TNF, macrophages stimulated with TNF (10 ng/ml). Bar graphs (mean ± SEM) represent quantitative analyses of qPCR or densitometry of the immunoblots from two independent experiments. Representative immunoblots are shown. Whole cell lysates from IFNγ macrophages were immunoblotted using antibodies to TCRζ, cleaved caspase 3 and β-actin, respectively. Quantitative densitometrical analyses of the immunoblots was normalized to β-actin.

The cytokine TNF is essential for host defense against mycobacteria and anti-TNF therapy may lead to disorganization of human tuberculous granuloma resulting in reactivation of latent tuberculosis [3], [16]–[19]. Since activation of the TCRαβ in macrophages results in CCL2 release, a key factor in granuloma formation, we examined whether TNFinhibition has a direct impact on the expression of the macrophage-TCRαβ. Uninfected or *BCG* infected macrophages were incubated in the presence of the anti-TNF antibody infliximab (50 µg/ml) or an isotype control antibody (anti-CD20, rituximab). Immunostaining revealed that TNF blockade inhibited TCRαβ expression relative to controls within 2h (Figure 7A). Consistent with this, the inhibitory effect could be completely reversed by re-exposure of macrophages to TNFfor 24 h indicating that macrophage-TCRαβexpression requires the presence of TNF (Figure S6A). TNF blockade also had an inhibitory effect on TCR β-chain mRNA expression (Figure 7B). Immunoblot revealed that TNF blockade not only suppressed expression of the TCRαβ ligand binding subunit but also that of the ζ-subunit (CD3) of the TCR signaling complex (Figure 7C). The latter is essential for TCRαβ stabilization on the cell surface [20] and its degradation is mediated by caspase 3 [21]. Immunoblot analysis and immunostaining showed that anti-TNF treatment results in an increase in cleaved caspase 3 in uninfected and *BCG* infected macrophages indicating that TNF blockade induces cleaved caspase 3 (Figure 7D, Figure S6B). Because TCRζ is required for stabilizing TCRαβ on the cell surface these findings identify TNF as a regulator of macrophage-TCRαβ expression.

### Loss of macrophage-TCR is associated with granuloma disorganization and reduced CCL2 expression in murine tuberculosis

**Figure 8:**
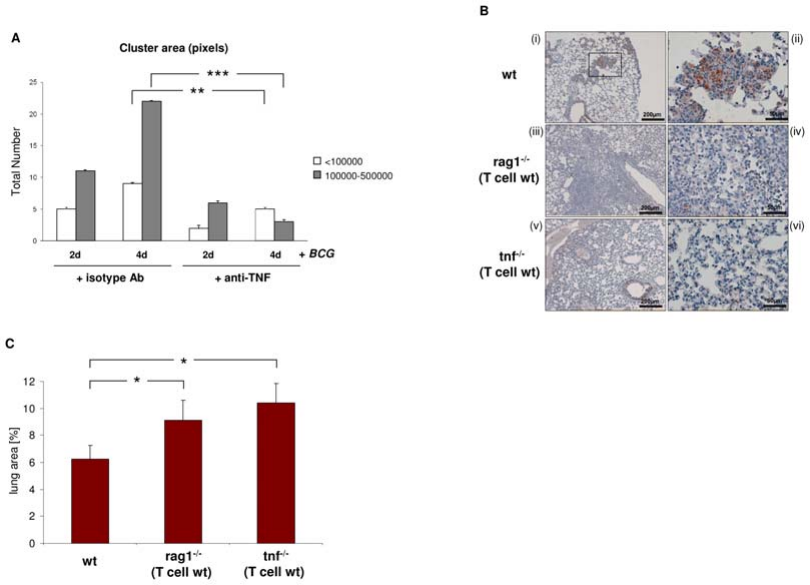
Loss of the macrophage-TCR in the presence of functional T cells results in disorganized granulomas and CCL2 suppression in murine pulmonary tuberculosis. (**A**) TNF blockade decreases the number and size of macrophage clusters induced by *BCG* infection *in vitro*. IFNγ macrophages were infected with *M. bovis BCG* in the presence of the anti-TNF antibody infliximab (50 µg/ml) or an equal amount of an isotype antibody (anti-CD20) for the indicated timepoints. Bar graphs represent total numbers of small (<100000 pixels) and large macrophage clusters (100000–500000 pixels) that were formed. The area (number of pixels) of the macrophage clusters was determined from electronic images. The results (mean ± SEM) are based on the analysis of three independent donors. ** p<0.01, ***p<0.001. (**B**) Lung sections of wildtype (wt) mice demonstrating formation of compact, well-circumscribed tuberculous lesions after four weeks infection with *M. tuberculosis* (i). A higher magnification of the boxed granuloma is shown in (ii). In contrast, chimeric rag1^–/–^(T cell wt) mice that lack the macrophage-TCR but have intact T cells develop disorganized tuberculous lesions that are consistently larger and more diffuse than those of wildtypes (iii, iv higher magnification). All sections were immunostained for CCL2. Note intense CCL2 staining (brown) in the tuberculous lesions of wt mice but near absence of CCL2 in the macrophage-TCR deficient rag1^–/–^(T cell wt) chimeras (i–iv). Chimeric tnf^–/–^(T cell wt) mice with systemic deletion of TNF but wildtype T cells display disorganized granulomas characterized by absence of CCL2 (v, vi) similarly as observed in macrophage-TCR deficient mice (iii, iv). All mice were infected via aerosol with ∼ 100 *M. tuberculosis* bacilli at the time of adoptive T cell transfer. The lung sections shown are representative of 5-7 mice in each experimental group. Scale bars are indicated. (**C**) Increased size of tuberculous granulomas in chimeric rag1^–/–^(T cell wt) and tnf^–/–^(T cell wt) mice relative to wildtype controls. Shown is the mean percentage of the lung area covered by granulomatous foci infection with *M. tuberculosis*. Data were calculated from scanned lung cross sections and are based on the analysis of five mice in each group. * p<0.05.

It is well-established that abrogation of TNF results in defective tuberculous granulomas [7], [8], [22]. Given this and our finding that *BCG* induces TCRαβ expression, we next tested whether TNF blockade affects *BCG* triggered macrophage cluster formation *in vitro.* We found that treatment with the anti-TNF antibody infliximab significantly reduced the number and size of human macrophage clusters that formed during infection with *M. bovis BCG in vitro* (Figure 8A). The presence of the TCRαβ in mouse macrophages offers the possibility to study the role of the TNF/ TCRαβ/ CCL2 regulatory axis we identified in human macrophages in tuberculosis *in vivo*. For this, we determined whether deletion of the macrophage-TCR impacts granuloma formation and macrophage CCL2 release in a murine model of *M. tuberculosis* infection. Wildtype (wt) mice (n = 5) and TCR deficient rag1^–/–^ mice (n = 7) that were reconstituted with wt CD3^+^ T cells were infected via aerosol with ∼100 *M. tuberculosis* bacilli [7]. The latter chimeric rag1^–/–^(T cell wt) mice develop lung tuberculosis in the presence of intact T cells but absence of TCR bearing macrophages and T lymphocytes are routinely detectable in the tuberculomas [8]. Four weeks post adoptive T cell transfer and *M. tuberculosis* infection all wt mice displayed compact, well-delineated granulomatous lesions in their lungs that were predominantly composed of macrophages and lymphocytes (Figure 8B i,ii). Rag1^–/–^(T cell wt) chimeras lacking the TCR in their macrophages developed granulomatous foci containing macrophages and lymphocytes as seen in the wildtype mice. However, these lesions were generally diffuse (Figure 8B iii,iv) and on average 1.5 fold larger than those of control mice indicating that rag dependent mechanisms outside the T cell system are required for proper granuloma formation in murine tuberculosis (Figure 8C). Importantly, immunostaining revealed abundant CCL2 staining in the tuberculous lesions of wt mice (Figure 8B i,ii) but CCL2 was routinely near absent in the chimeras lacking the macrophage-TCR (Figure 8B iii,iv). Collectively, these results demonstrate *in vivo* that ablation of the variable macrophage immune receptor in murine lung tuberculosis is associated with suppression of CCL2 and defective granulomas formation.

We finally tested the impact of TNF abrogation on CCL2 expression and tuberculoma formation in TNF deficient mice that were reconstituted with wildtype CD3^+^ T cells (TNF^–/–^(T cell wt) mice). Infection with *M. tuberculosis* infection in these chimeras resulted in disorganized tuberculous granulomas in which CCL2 was consistently absent (Figure 8B v,vi). Similarly as in the rag1^–/–^(T cell wt) chimeras, the granulomatous foci in TNF^–/–^(T cell wt) mice displayed a consistent increase in size (1.7 fold vs. controls) (Figure 8C). This together with our observation that TNF blockade inhibits expression of the macrophage-TCR strongly suggests that an intact TNF/TCR macrophage pathway is required for CCL2 production in the tuberculous granuloma. Consistent with this, we found no CCL2 expression in the tuberculous lymphnode of a patient who received therapeutic anti-TNF treatment (adalimumab) (Figure S7).

## Discussion

In this study, we report the existence of an as yet unrecognized recombinatorial TCRαβ based immune receptor in monocytes/ macrophages (macrophage-TCRαβ) and provide evidence for its implication in a major infectious disease - tuberculosis. We find that monocytes and macrophages have rearranged Vβ gene loci and consistently express diverse TCR Vαβ repertoires and show that macrophage-TCRαβ engagement induces the release of the monocyte chemoattractant CCL2 and demonstrate that the expression of the novel variable immune receptor depends on TNF. Furthermore, blockade of TNF in macrophages results in caspase 3 cleavage, TCRζ degradation and TCRαβ downregulation.

Traditionally, variable immune receptors are thought to be restricted to cells of lymphoid origin [1]. However, recent work from our laboratories and others demonstrating TCRαβ and TCRγδ expression in neutrophils and eosinophils [9]–[11], antigen receptor gene rearrangement in thymic granulocytes of mice [23], and NK cells which display immunological memory in response to viral infection [24] challenge this longstanding concept. These findings, which identify lymphoid features in myeloid cells, and the reciprocal demonstration that lymphocyte progenitors retain myeloid potential [25] and B cells in primitive vertebrates possess phagocytotic capabilities [26] extend the current concept of the strict dichotomy of the vertebrate immune system into non-specific/ myeloid and antigen-specific/ lymphoid immunity [1] and suggest a closer kinship between both lineages than commonly appreciated.

**Figure 9:**
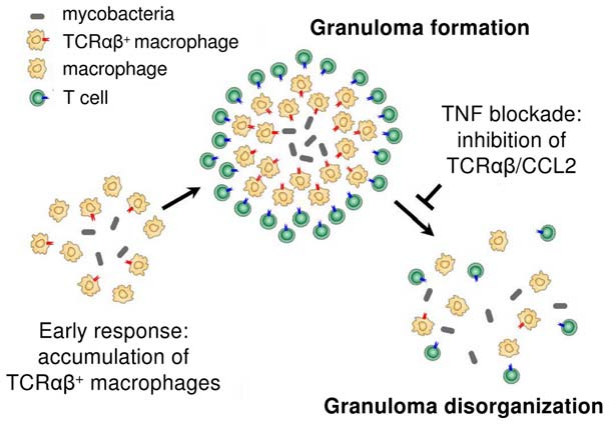
Proposed role of the macrophage recombinatorial TCRαβ in the formation of the tuberculous granuloma and its regulatory interactions with TNF and CCL2.

Combined *in vitro* evidence demonstrates a role for the macrophage-TCRαβ in the modulation of phagocytosis and the macrophage response during the initial phase of mycobacterial infection. *In vivo*, we find that the host/pathogen interface of human pulmonary tuberculomas is characterized by the massive presence of TCRαβ bearing macrophage-derived epithelioid cells and deletion of the macrophage-TCR in murine tuberculosis is associated with disorganized granuloma structure. Murine studies have previously suggested the possibility that variable immune receptors oustide the realm of T cells are involved in the control of mycobacterial infection [6]–[8]. Our demonstration that a TNF-dependent variable macrophage immune receptor is part of the host defense against mycobacteria provides evidence for this concept. In particular, our results suggest that macrophage based antigen-specific host response mechanisms are operative in the development of the tuberculous granuloma and thus add an all new aspect to the current understanding of tuberculoma formation (Figure 9). Furthermore, since it is well established that TNF is indispensable for the proper formation of tuberculous granulomas [7], [8], [22], [27], the finding that TNF blockade leads to suppression of the macrophage-TCRαβ provides a novel potential mechanism that may underly the reactivation of tuberculosis during therapeutic anti-TNF treatment [5].

TNF dependent regulation of the macrophage-TCR implies the involvement of the TNF receptor 1 and/or TNF receptor 2 signaling pathways in the control of expression of the novel immune receptor. At this point, it is unclear at which level the TNF receptor signaling cascade interferes with the macrophage-TCR machinery. It will be challenging to identify signaling components that connect TNF to the TCR in macrophages. Also, it will be interesting to see if TCR activation has an impact on TNF expression.

Given the near-ubiquitous presence of macrophages and their involvement in acute and chronic inflammation, it is likely that the macrophage-TCRαβ is implicated in further pathologies beyond tuberculosis. Candidate diseases include chronic inflammatory diseases such as atherosclerosis and autoimmune disorders such as rheumatoid arthritis. Efforts are underway to further explore the biological function of the recombinatorial macrophage receptor in the immune response.

## Materials and Methods

### Isolation and culturing of monocytes/macrophages

Human monocytes/macrophages were isolated as previously reported [28] and purified by CD14^+^ Magnetic Cell Sorting (MACS) (Miltenyi Biotec, Bergisch Gladbach, Germany) Monocyte cell purity was routinely >99.5% as assessed by flow cytometry (Figure S1A) using the following antibodies: anti-CD14-FITC (DAKO, Glastrup, Denmark), anti-CD3-PE (BD Biosciences, Franklin Lakes, USA), anti-CD2-PE (BD Biosciences), mouse IgG1-FITC Isotype control and mouse IgG1-PE Isotype control. CD14^+^ monocytes were cultivated for 6 days in X-VIVO 10 serum-free and endotoxin-free medium (Cambrex, Verviers, Belgium) at a concentration of 5×10^5^ cells/ml in the presence of IFNγ (1000 U/ml) (PeproTech, Rocky Hill, USA) or IL-4 (10 ng/ml) (Tebu Bio, Frankfurt, Germany) to induce differentiation into Th1 and Th2 polarized macrophages. X-VIVO 10 is an optimal medium for cultivating human monocyte-derived macrophages [12], [28] which does not contain exogenous growth factors or artificial stimulators of cellular proliferation. Burst-forming unit-erythroid (BFU-E) and colony-forming units containing granulocytes and macrophages (CFU-GM) were generated from human CD34 progenitor cells as previously described [29].

### Ethics statement

Infected granuloma tissue was obtained from the Department of Pathology, Universitätsmedizin Göttingen, Georg-August-University. Bronchial alveolar lavage fluid samples were excess material derived from three patients in the course of their medical care. The use of these specimens and mononuclear cells from healthy probands was approved by the Ethics Committee of the Faculty of Medicine Mannheim, University of Heidelberg, Germany and Ethics Committee of the Medical University of Göttingen, Germany. (Permit Number: 2007-254N-MA and 27/6/11). All patient samples were stricly anonymized. In accordance with the Declaration of Helsinki [30] no written informed consent was provided by study participants and/or their legal guardians.

The study was carried out in strict accordance with the recommendations in the Guide for the Care and Use of Laboratory Animals of the National Institutes of Health. Mouse procedures performed in this study were conducted at the Centenary Institute, after protocol review and approval by the University of Sydney Animal Ethics Committee (K75/3-2004/3/3878) and at the animal facility of the Klinikum Göttingen Georg-August-Universität (Göttingen, Germany) according to the Deutsche Tierschutzgesetz (LAVES Niedersachsen A-008/09), after protocol review and approval by the University of Göttingen. All surgery was performed under sodium pentobarbital anesthesia, and all efforts were made to minimize suffering by the attending veterinarian.

### Animal models

C57BL/6 mice and rag1 null mutant mice (rag1^–/–^) were bred and maintained at the animal facility, Klinikum Göttingen Georg-August-Universität (Göttingen, Germany). Macrophages from the spleen were collected and purified by CD11b-MACS for further analyses. Thioglycollate-elicited mouse peritoneal macrophages were collected according to standard protocols. Mice used in the *M. tuberculosis* infection experiments were maintained under specific pathogen-free conditions in the Animal Facility of the Centenary Institute of Cancer Medicine and Cell Biology (Newtown, Australia). Genetically modified chimeric mice were generated by transfer of 1×10^6^ purified wildtype CD3^+^ T cells into TCR deficient rag1^–/–^ mice and TNF null mice, respectively (n = 7 in each group) following whole body irradiation with 5 Gy as previously described [7]. At the time of T cell reconstitution wildtype, rag1^–/–^(CD3 rag1^+/+^) and TNF^–/–^(CD3 TNF^+/+^) chimeric mice, respectively, were infected via aerosol with ∼ 100 *M. tuberculosis* bacilli (H37Rv) utilizing a Middlebrook airborne infection apparatus. After four weeks all mice were sacrificed and paraffin embedded lung sections were used for immunohistochemical analyses.

### Flow cytometry

PBMC were isolated from freshly collected whole blood or buffy coats from healthy individuals by Ficoll density gradient centrifugation as previously described [9]. Cells were first fixed with 4% paraformaldehyde and then blocked with normal horse serum (Jackson Immuno Research Laboratories). TCR staining was performed using the TCRβF1 antibody (clone 8A3), which was also utilized in the immunocytochemistry and immunoblot experiments. This framework antibody recognizes TCRαβ dimers. Mouse IgG1 and IgG2b antibodies serve as isotype controls and CD235a (purified) as a nonsense control antibody (all BD Bioscience). Antibody binding was detected with a secondary goat-anti-mouse FITC-labeled antibody (BD Bioscience). After saturating with normal mouse serum (Jackson Immuno Research Laboratories) the cells were stained with the following directly labeled antibodies: CD45 APC-H7, CD3 PerCP-Cy5.5, CD8-PE and CD14-APC (BD Bioscience). Analyses were performed on a FACS Canto using the FACS Diva software (BD Bioscience).

### Laser scanning cytometry (LSC)

Monocytes/macrophages were stained with mouse anti-human TCRαF1/TCRβF1 antibodies (Thermo Scientific, Waltham, USA) in combination with Alexa Fluor 555 goat anti-mouse IgG (Invitrogen, Carlsbad, USA) and analyzed on an iCYS laser scanning cytometer (Compucyte, Cambridge, MA, USA). For assessment of apoptosis, annexin V/ PI staining was used. Contouring of cells was achieved by nuclear staining with DAPI. Photomultiplier tube settings for voltage, offset and gain were optimized (Figure S1C). Data were acquired and analyzed using the iCYS cytometric acquisition and analysis software (CompuCyte). For statistical analysis, the entire area of the microscopic slide was scanned and for every event pictures of each channel (red, blue, and scatter) were recorded and merged within a gallery. The count settings selected for signal area of DAPI staining were within the range 15–150 µm^2^ in order to exclude artefacts such as debris.

### Protein isolation and immunoblotting

Modified RIPA buffer was used to extract whole cell lysates. SDS-PAGE and transfer to nitrocellulose membranes was conducted utilising the NuPAGE protein electrophoresis systems (Invitrogen). The primary monoclonal mouse anti-human TCRαF1/TCRβF1 antibodies (Thermo Scientific) and the polyclonoal antibodies for cleaved caspase 3 (ASP175) or CD3 zeta (Abcam, Cambridge, UK) were used. Mouse monoclonal antibodies to β-actin (Abcam) were used as loading control.

### Protein identification by MALDI-TOF mass spectrometry

Immunoprecipitated TCRαβ bands were separated by SDS-PAGE and visualized by silver staining. The predicted 58kD single band was excised and destained. An in-gel digestion with trypsin was performed according to a standard protocol [31]. After o/n incubation at 37°C, peptides were extracted with a C18 affinity chromatography (ZipTip, Millipore) and eluted with 0.5% formic acid in 1∶1 (v/v) water: acetonitrile. The eluate (1 µl) was mixed with saturated solution (9 µl) of α-cyano-4-hydroxycinnamic acid in 50% CAN and 0.1% TFA, spotted onto a steel target and the droplet was air dried prior to MS-analysis. Peptide mass fingerprinting was performed on a MALDI-TOF-MS (Autoflex II, Bruker Daltonics) operating in the reflector mode. The MS spectra for peaks in the range of 1-3.5 kDa were generated by summarizing 350 laser shots (50 laser shots at 7 different spot positions). Spectra were analyzed using the flexanalysis software (Bruker) [31], [32]. Analysis of the MS spectra was performed utilizing the BioTools software (Bruker) in combination with an integrated online link to the Mascot database (www.matrixscience.com).

### TCRβ locus recombination assay

DNA from 10^6^ monocytes, IFNγ-macrophages, HepG2 (negative controls) and PBMC (positive controls) was isolated using the Wizard Genomic DNA purification Kit (Promega). Screening for Dβ → Jβ and Vβ → Jβ rearrangements at the TCRβ locus was performed by PCR utilizing a modified non-multiplex approach according to the protocols by van Dongen et al. [13] and confirmed by sequencing. The customized primers used can be requested by the authors.

### TCR CDR3 length spectratyping and qPCR

RNA from all monocyte/macrophage populations was prepared with TRI Reagent (Sigma) and transcribed into cDNA using the Reverse Transcription System (Promega). RT-PCR expression profiling of components of the TCR machinery and size spectratyping of the antigen binding TCR Vα/Vβ CDR3 regions were performed as previously reported [9]. Vαβ spectratypes of the human TCR CDR3 regions were assessed on a CEQ™ 8000 Genetic Analysis System (Beckman Coulter) using the D4-labeled primers D4-GCAGACAGACTTGTCACTGG (TCRα) and D4-TTGGGTGTGGGAGATCTCTGC (TCRβ), respectively. To determine the detailed CDR3 clonotypes for Vβ13a, specific RT-PCR amplification products were cloned into a pCR-TOPO vector (TOPO TA Cloning Kit, Invitrogen) using standard protocols. The cDNA sequences of the Vβ13a CDR3 regions were analyzed from at least 10 randomly picked clones. qPCR for the TCRβ constant chain and CCL2 was conducted using the IQ SYBR Supermix (Biorad, Hercules, USA). B2MG and GAPDH were used as housekeeping genes. Purity of monocyte/macrophage RNA was confirmed by PCR amplification of the leukocyte lineage markers CD2, CD8, CD14, CD64, CD68, CD163, MMP25 and MPO, respectively (exemplified in Figure 2D). Authenticity of all relevant PCR products was confirmed by sequencing. PCR runs were repeated at least twice. The sequences of additional PCR primers used in this study can be requested from the authors.

### Immunocyto/-histochemistry

Before immunostaining all human and mouse tissue sections were deparaffinized and rehydrated. For immunostaining, 5 µm tissue sections or cells cultivated on coverslips were blocked with 5% goat serum in PBS (1% BSA), incubated with a combination of primary antibodies at 4°C overnight, washed in PBS for 15 min, and incubated with a combination of appropriate secondary antibodies. The following antibodies were used: FITC-labeled mouse anti-human CD68 (KP1, 1∶50) (DakoCytomation), mouse anti-human antibodies to TCRαF1 (clone 3A8) and TCRβF1 (clone 8A3) (1∶100, Thermo Scientific), hamster anti-mouse TCRβ (clone H57-597) (1∶50, BD Biosciences), mouse anti-human MHC Class II (clone 910/D7, 1∶200) (Acris Antibodies), anti-mouse F4/80 (AbD Serotec), rabbit anti-human CD163 (Santa Cruz), anti-mycobacterium tuberculosis-FITC (Acris, Herford, Germany), rabbit anti-human CD2 (Thermo Scientific) and anti-cleaved caspase 3 (ASP175) (Cell Signaling Technology, Beverly, USA). Goat anti-mouse IgG, Alexa 488 (Invitrogen), donkey anti-rabbit IgG, Cy3 (Dianova) (both 1∶400), goat anti-hamster IgG, Alexa 488, donkey anti-rat IgG, Cy3 (Jackson ImmunoResearch) and goat anti-mouse IgG, Alexa 555 (MoBiTec, Göttingen, Germany) were used as secondary antibodies. Mouse IgG1 and IgG2a (BD Biosciences), rat IgG2 (Biozol) and hamster IgG2 isotype control antibodies (BD Biosciences) were used as negative controls. For fluorescence imaging DRAQ5 (1∶2500) (eBioscience) and Vectashield Mounting Medium with DAPI (Vector Laboratories), respectively, were used for nuclear staining. Positive staining was either visualized by a Leica DMIRE2 microscope and the FW400 software or a Leica TCS SP-2 laser-scanning spectral confocal microscope equipped with a 63×1.32 objective (Leica Microsystems). Excitation sources were an argon laser (488 nm), a crypton laser (568 nm) and a helium/neon laser (633 nm). Data were acquired and analyzed using the Leica confocal software. Two- and three-color images were acquired using a sequential scan mode [28]. For light microscopy, samples were incubated with the TCRα/TCRβ antibodies, rabbit antibodies to CCL2 (ab7202, Abcam, Cambridge, UK) or rabbit anti-human CCL2 (ab9669, Abcam), respectively, in combination with Envision+ system-HRP anti-mouse or anti-rabbit HRP (Dako). Quantitation of TCRαβ^+^ cells in immunostained cytospin preparations and tissue sections was conducted by at least two blinded assessors and subjected to statistical analysis. For quantitation of electronic fluorescence microscopy images the NIH image J software was used.

### Immunoelectron microscopy

IFNγ stimulated macrophages were incubated in the presence or absence of *BCG* for 6 h and subsequently fixed in 4 % formaldehyde and 0.2 % glutaraldehyde (0.1 M phosphate buffer). After washing the cells were scraped from the dish in 0.1 M phosphate buffer containing 1% gelatin, spun down and resuspended in 10% gelatin (0.1 M phosphate buffer) at 37°C. The cooled gelatin pellets were cut in small blocks, infiltrated in 2.3 M sucrose in 0.1 M phosphate buffer and mounted onto aluminum pins for ultramicrotomy before shock freezing. Ultrathin cryosections were picked in a 1∶1 mixture of 2 % methylcellulose and 2.3 M sucrose. For immuno-labeling sections were incubated with monoclonal antibodies specific for TCRαF1 (Thermo Scientific) and rabbit anti-mouse IgG antiserum (Rockland, Gilbertsville, PA, USA) followed by protein A-gold (10 nm). Alternatively, the primary mouse antibody was detected utilizing goat anti-mouse antibodies conjugated to gold (Aurion, Wageningen, The Netherlands). Sections were analyzed using a LEO EM912 Omega transmission electron microscope (Zeiss, Oberkochen, Germany) and digital micrographs were obtained with an on-axis 2048×2048-CCD camera (Proscan, Scheuring, Germany).

### Laser microdissection

CD68^+^/TCRαβ^+^ cells were identified in 2 µm lung sections from patients with pulmonary tuberculosis by immunofluorescence microscopy (Carl Zeiss Microimaging, Göttingen, Germany). Single double positive cells or small cell clusters were then microdissected using a P.A.L.M. Laser Microdissection System with laser pressure catapulting (LPC) (*P.A.L.M.* Microlaser Technologies, Bernried, Germany). Total RNA from clusters of 20–30 cells was isolated using the invisorb RNA kit I (Invitek, Berlin, Germany) and cDNA was synthesized (Superscript III First-Strand cDNA Synthesis Kit, Invitrogen, Carlsbad, USA).

### Infection with *M. bovis BCG* and quantitation of *BCG* phagocytosis

Attenuated *Mycobacterium bovis BCG* (Bacillus Calmette-Guérin, BCG-medac) bacteria, strain RIVM, were re-constituted with the supplied 0.9% NaCl solvent (*BCG*-Medac, Hamburg, Germany) and used for infection of macrophages (MΦ:*BCG*  =  1∶10). For quantitation of phagocytosed bacilli, *BCG* mycobacteria were immunostained with a FITC-labeled antibody to *M. tuberculosis* (Acris). *BCG* phagocytosis was quantitated from a total of 20 randomly selected fluorescence microscopy images using the NIH image J software. The phagocytotic index (PI) was calculated as (percentage of macrophages containing at least one bacterium) x (mean area of bacterial staining per cell).

### Assessment of *BCG*/ macrophage cluster areas

The areas (number of pixels) of the *M. bovis BCG* infected macrophage clusters were determined from electronic images using the image J software. From each individual 30 clusters were analyzed.

### Clonotype analysis of *M. bovis BCG* infected macrophages

Isolated CD14^+^ cells were allowed to differentiate into macrophages for 6 days on semisolid agarose plates (0.4% agarose in X-VIVO 10 medium) in the presence of IFNγ (1000 U/ml) and *M. bovis BCG*. Single foci were collected using a 1 ml syringe containing 100 µl PBS. cDNA was prepared using the RNeasy MircoKit and Sensiscript RT Kit (Qiagen, Hilden, Germany) and subjected to TCR Vβ CDR3 spectratyping.

### Cytokine analysis

For selective activation of the TCR, freshly obtained CD14^+^ monocytes from two healthy donors were allowed to differentiate into macrophages in the presence of IFNγ. After 6 days IFNγ-polarized macrophages were washed twice with X-VIVO-10 medium (Cambrex) and co-stimulated with an endotoxin-free mouse anti-human CD3 (1 µg/ml, Beckman Coulter) monoclonal antibody as previously reported [9]. The culture supernatants were collected at various timepoints and the release of a selected panel of cytokines, chemokines and growth factors (n = 15) (Table S1) was assessed utilizing a customized Luminex-based multiplex Procarta Cytokine Assay Kit (Multimetrix, Heidelberg, Germany). All measurements were conducted at least in duplicate. In addition, CCL2 (Thermo Scientific) and CCL5 (Qiagen) in the supernatants were quantified using specific ELISAs.

### Bead p**hagocytosis assay**

For phagocytosis of baits targeted to the TCRαβ, IFNγ-polarized macrophages were co-incubated with polystyrene bead baits (Ø 4.5 µm, Invitrogen) coated with anti-TCRα/anti-TCRβ, anti-CD11b (BD Biosciences), nonspecific IgG isotype control antibodies or albumin for 15 min, 1 h and 10 h, respectively, at 37°C (MΦ:beads  =  1∶1, 5 µg protein/10^7^ beads). In addition, phagocytosis of albumin-coated beads was assessed in the presence of uncoupled anti-TCRα/anti-TCRβ antibodies. Quantitation of phagocytosed beads was conducted by bright field microscopy of at least 12 randomly selected fields of vision performed by two independent observers.

### Statistical analyses

Student’s *t* test was used to compare the significance of differences between groups. Results were expressed as means ± SD. *p*<0.05 was considered statistically significant.

### Accession numbers

#### Human genes

TCRα-VJ: AE000660, AE000662, TCRα-constant: X02883, TCRβ: L36092, CD3ζ: NM_198053.2, ZAP70: NM_001079, LAT: NM_014387, Fyn: NM_153048, Lck: NM_001042771, CD2: NM_001767, CD8: NM_001768, CD14: NM_000591, CD68: NM_001040059, CD163: NM_004244, GAPDH: NM_002046.3, CCL2: NM_002982.3.

#### Human proteins (Swiss-Prot)

TCRα: P01848, TCRβ: P01850, TNFα : P01375, CCL2 : P13500, CCL5 : P13501, CD14: P08571, CD3: P07766, CD2: P06729, CD68 : P34810, CD163 : Q86VB7, CR3: P11215, MHC-II : P04232, IFNγ: P01579, IL-4: P05112, β-actin : P60709.

#### Murine genes

TCRα: M64239, TCRβ: M26053, M26057, TCRβ-V: NG_006980.

#### Murine proteins (Swiss-Prot)

TCRα: M64239, TCRβ: M26057; M26053, Rag1: AAP76401, F4/80: Q3S4B0, TNFα:P06804.

## Supporting Information

Figure S1 **ΤCRαβ expression by subpopulations of human monocytes/macrophages. (A)** CD14+ cells isolated from whole blood of healthy donors used in all experiments were routinely >99.5 % pure before differentiation into macrophages was induced. Shown is a representative flow cytometric analysis using the lineage markers CD2, CD3 and CD14, respectively. PBMC are shown as reference (left). **(B)** Isotype antibody (mouse IgG2b) and irrelevant antibody (CD235a) used as negative controls in flow cytometric analysis of TCRβ expression. CD14+ monocytes are in pink color, CD3+ lymphocytes in blue. CD235a, glycophorin A. **(C)** LSC gallery of naïve macrophages (CD14) and IL-4 or IFNγ activated macrophages immunostained for TCRαβ (red). The iCYS image gallery depicts 25 examples of individual events with the event of interest in the center of the image. Note that the scanning cytometer has a broad focal plain to account for variation in cell morphology on a flat surface. The count setting protocol used for iCYS event collection is indicated. For quantitation single cells were directed to a dot plot of blue (DAPI) vs. orange (TCRαβ) probe MaxPixel. TCRαβ+ cells were identified by setting a single gate based on orange fluorescence in the reference coverslip on which macrophages from healthy donors were grown. Black sections mark boundaries of analyzed areas. Monocytes from a healthy donor were cultured on glass coverslips for 6 days in the presence or absence of IL-4 and IFNγ, respectively, and subsequently stained with Alexa555-labeled antibodies to TCRαβ. **(D)** Immunocytochemical double-staining demonstrating the presence of the TCRαβ in normal human BAL macrophages (patient 2, 71 y, male). The merged confocal images show ΤCRαβ (green)/CD163 (red) double positive alveolar macrophages. A close-up view of the outlined area is shown in the right panel. Nuclei (blue), DRAQ5. Giemsa-staining of the BAL cytospin preparation is shown in the left panel. *KP*, *K. pneumoniae*. The patient showed no clinical signs of pneumonia. **(E)** Immunocytochemical staining demonstrating the presence of the TCRαβ (green) in normal human BAL macrophages (patient 3, 59 y, female). A close-up view of the outlined area is shown in the right panel. The arrow points to a TCRαβ+ alveolar macrophage. Nuclei (blue), DRAQ5.
(TIF)

Figure S2 **(A) The monocyte/macrophage TCRαβ is a recombinatorial receptor.** Quantitative synopsis of the CDR3 length variants in three individuals. Global analyses of the expressed TCR Vα and Vβ chain CDR3 length repertoires (Vα1-20; Vβ1-25) in individuals 1- 3 reveal increased repertoire diversity in IFNγ activated macrophages relative to monocytes and IL-4 macrophages. **(B)** Detailed Vβ repertoires expressed by additional CFU-GM progenitor colonies from donors A and B.
(TIF)

Figure S3 **Effect of CD3 mediated TCR activation on cytokine release from macrophages.** Aliquots of 5×105 IFNγ macrophages were incubated with soluble antibodies to CD3, isotype control antibodies (I) or in the absence of antibodies (-) for the indicated timepoints as in Figure 3B. Cytokines were determined in the supernatant by multiplex cytokine assay. The results are summarized in Table S1. Macrophages were collected from two healthy donors (ind 1, ind 2).
(TIF)

Figure S4 **Infection of macrophages with** ***M. bovis BCG*** **induces TCRαβ expression** ***in vitro*** **. (A)** Confocal image of a TCRαβ expressing macrophage cluster induced by infection with *BCG*. Uninfected IFNγ macrophages from the same donor (donor B) are shown left. IFNγ macrophages were incubated in the presence or absence of FITC-labeled *BCG* for 6 days. White arrows highlight TCRαβ+ macrophages. **(B)** TCR Vβ repertoire analysis of randomly selected *BCG*/macrophage clusters from donor B reveals expression of highly restricted TCR Vβ chain repertoires. *BCG*/macrophage clusters 1-5 were subjected to RT-PCR and CDR3 spectratyping. The identified TCR Vβ repertoires are shown for each individual cluster. Note that next to the Vβ1 only few additional Vβ chains are expressed. The single peaks are indicative of monoclonality.
(TIF)

Figure S5 **Suppression of macrophage-TCRαβ expression in the tuberculous granulomas of a patient receiving anti-TNF therapy. (A)** Isotype control staining of the macrophage markers CD68 and CD163 was performed using the same staining conditions as in Figure 6B. Bars in both images span the inner hostpathogen contact zone. N, necrotic caseous core. 40x. **(B)** Immunofluorescence microscopy of two tuberculous granulomas present in the lymph node of a patient with therapeutic anti-TNF treatment (adalimumab). Paraffin sections of the granulomas were double-stained for TCRαβ Alexa 555, red) and the macrophage marker CD68 (FITC, green). Nuclei are DAPI-stained (blue). Shown are merged images. Scale bars are indicated. Note the consistent absence of TCR bearing macrophages (TCRαβ+/CD68+, yellow) within the granulomas. The AFB (Acid.Fast Bacilli) test was used to confirm active infection with acid fast mycobacteria in the patient.
(TIF)

Figure S6 **TNF blockade inhibits expression of the macrophage-TCRαβ. (A)** Re-exposure to TNF reverses macrophage-TCR suppression induced by TNF blockade. IFNγ activated macrophages were co-cultured in the presence of *M. bovis BCG* for 24 h followed by incubation with the monospecific anti-TNF antibody infliximab (50 µg/ml) for 2 h and 24 h, respectively. Anti-TNF treatment potently inhibits macrophage-TCR expression (red, Alexa-555 labeled) already after 2 h (left panel). TNFstimulation 10 ng/ml) of anti-TNF treated macrophagesfor 24 h induces TCR expression (right panel). The results shown are representative of two independent experiments. **(B)** Immunofluorescence staining demonstrates the induction of cleaved caspase 3 (green) in uninfected and *M. bovis BCG* infected macrophages by TNF blockade. Human CD14+.
(TIF)

Figure S7 **Suppression of macrophage CCL2 expression in the tuberculous granulomas of a patient receiving anti-TNF therapy.** Light microscopic immunostaining reveals near absence of CCL2 (DAB, brown) from the lymph node of a patient with therapeutic anti-TNF treatment (adalimumab) (right). A lymph node from an untreated patient displaying intense CCL2 staining is shown as reference (left). Top panel, 10x; the highlighted areas are shown at 63x magnification (bottom).
(TIF)

Table S1 **Synopsis of cytokine/chemokine/growth factor release by IFNγ macrophages.**
(PDF)

Table S2 **Histological and immunohistochemical features of granulomas from patients with pulmonary tuberculosis.**
(PDF)
