# Supplementary figures and images for: A TNF-Regulated Recombinatorial Macrophage Immune Receptor Implicated in Granuloma Formation in Tuberculosis
Source: PLoS Pathog. 2011 Nov 17;7(11):e1002375. doi: 10.1371/journal.ppat.1002375 (PMC3219713; doi:10.1371/journal.ppat.1002375)

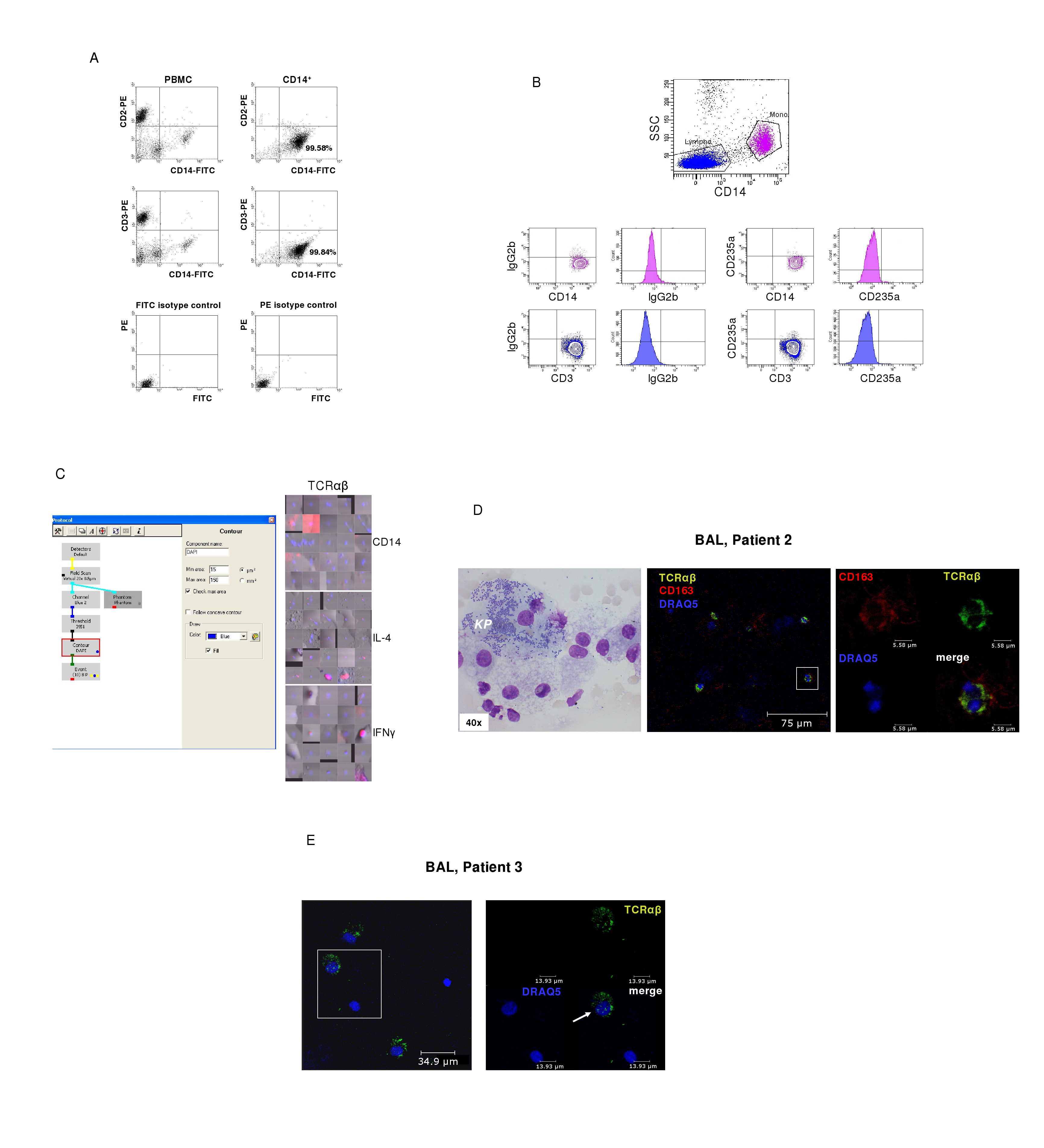

Supplement: Figure S1 — ΤCRαβ expression by subpopulations of human monocytes/macrophages. (A) CD14+ cells isolated from whole blood of healthy donors used in all experiments were routinely >99.5 % pure before differentiation into macrophages was induced. Shown is a representative flow cytometric analysis using the lineage markers CD2, CD3 and CD14, respectively. PBMC are shown as reference (left). (B) Isotype antibody (mouse IgG2b) and irrelevant antibody (CD235a) used as negative controls in flow cytometric analysis of TCRβ expression. CD14+ monocytes are in pink color, CD3+ lymphocytes in blue. CD235a, glycophorin A. (C) LSC gallery of naïve macrophages (CD14) and IL-4 or IFNγ activated macrophages immunostained for TCRαβ (red). The iCYS image gallery depicts 25 examples of individual events with the event of interest in the center of the image. Note that the scanning cytometer has a broad focal plain to account for variation in cell morphology on a flat surface. The count setting protocol used for iCYS event collection is indicated. For quantitation single cells were directed to a dot plot of blue (DAPI) vs. orange (TCRαβ) probe MaxPixel. TCRαβ+ cells were identified by setting a single gate based on orange fluorescence in the reference coverslip on which macrophages from healthy donors were grown. Black sections mark boundaries of analyzed areas. Monocytes from a healthy donor were cultured on glass coverslips for 6 days in the presence or absence of IL-4 and IFNγ, respectively, and subsequently stained with Alexa555-labeled antibodies to TCRαβ. (D) Immunocytochemical double-staining demonstrating the presence of the TCRαβ in normal human BAL macrophages (patient 2, 71 y, male). The merged confocal images show ΤCRαβ (green)/CD163 (red) double positive alveolar macrophages. A close-up view of the outlined area is shown in the right panel. Nuclei (blue), DRAQ5. Giemsa-staining of the BAL cytospin preparation is shown in the left panel. KP, K. pneumoniae. The patient showed no clinical s [file ppat.1002375.s001.tif]

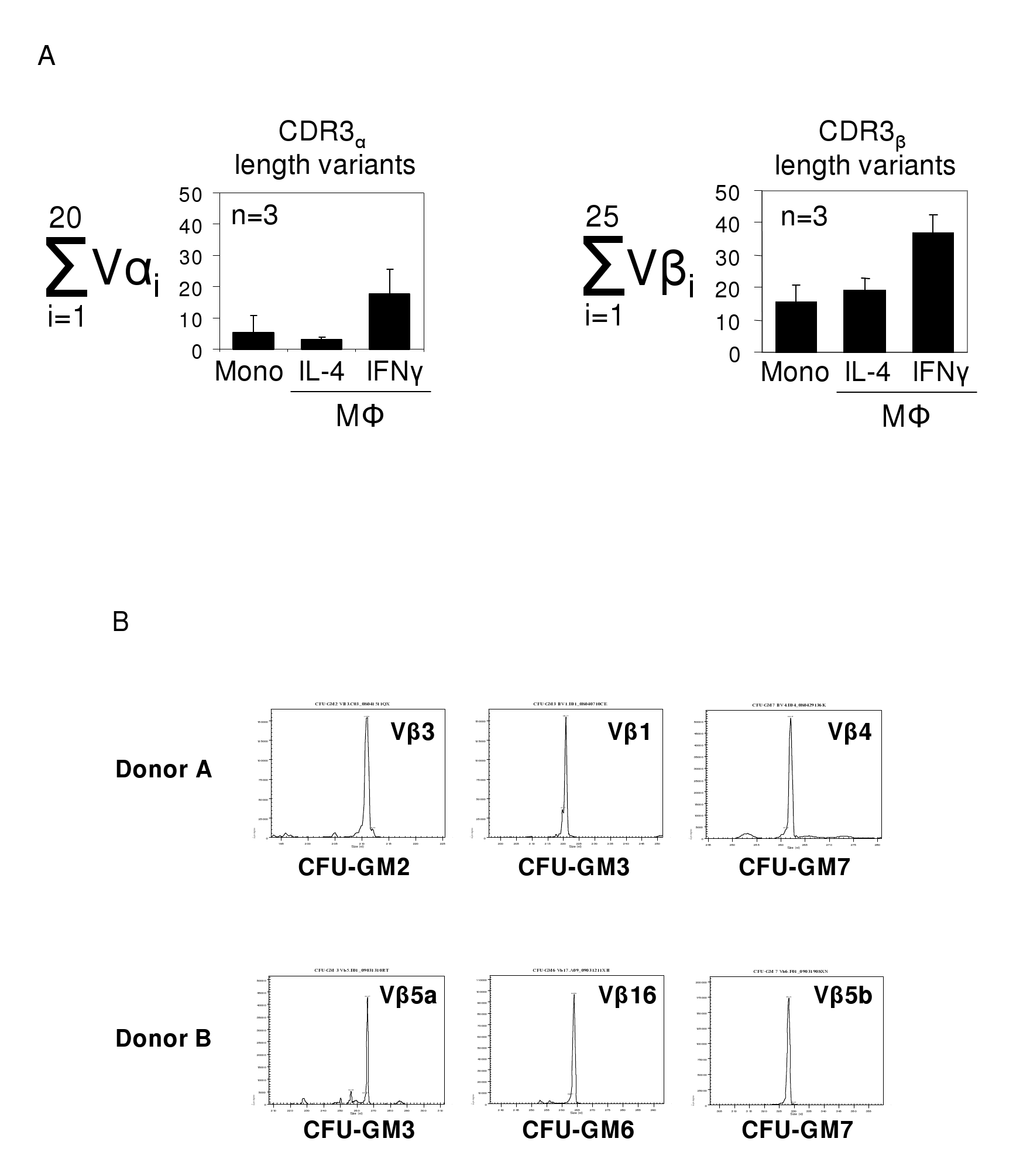

Supplement: Figure S2 — (A) The monocyte/macrophage TCRαβ is a recombinatorial receptor. Quantitative synopsis of the CDR3 length variants in three individuals. Global analyses of the expressed TCR Vα and Vβ chain CDR3 length repertoires (Vα1-20; Vβ1-25) in individuals 1- 3 reveal increased repertoire diversity in IFNγ activated macrophages relative to monocytes and IL-4 macrophages. (B) Detailed Vβ repertoires expressed by additional CFU-GM progenitor colonies from donors A and B. (TIF) [file ppat.1002375.s002.tif]

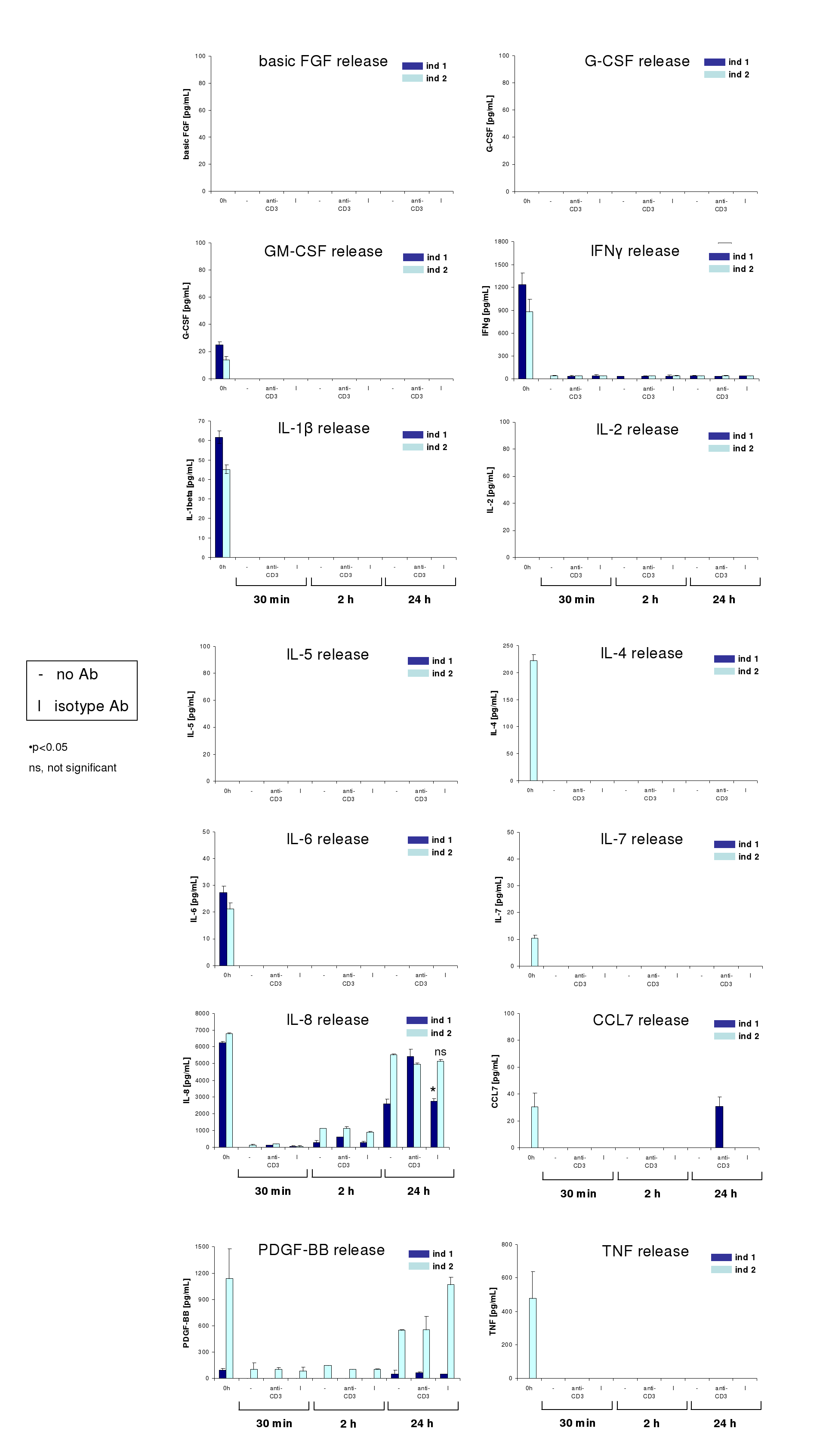

Supplement: Figure S3 — Effect of CD3 mediated TCR activation on cytokine release from macrophages. Aliquots of 5×10^5^ IFNγ macrophages were incubated with soluble antibodies to CD3, isotype control antibodies (I) or in the absence of antibodies (-) for the indicated timepoints as in Figure 3B. Cytokines were determined in the supernatant by multiplex cytokine assay. The results are summarized in Table S1. Macrophages were collected from two healthy donors (ind 1, ind 2). (TIF) [file ppat.1002375.s003.tif]

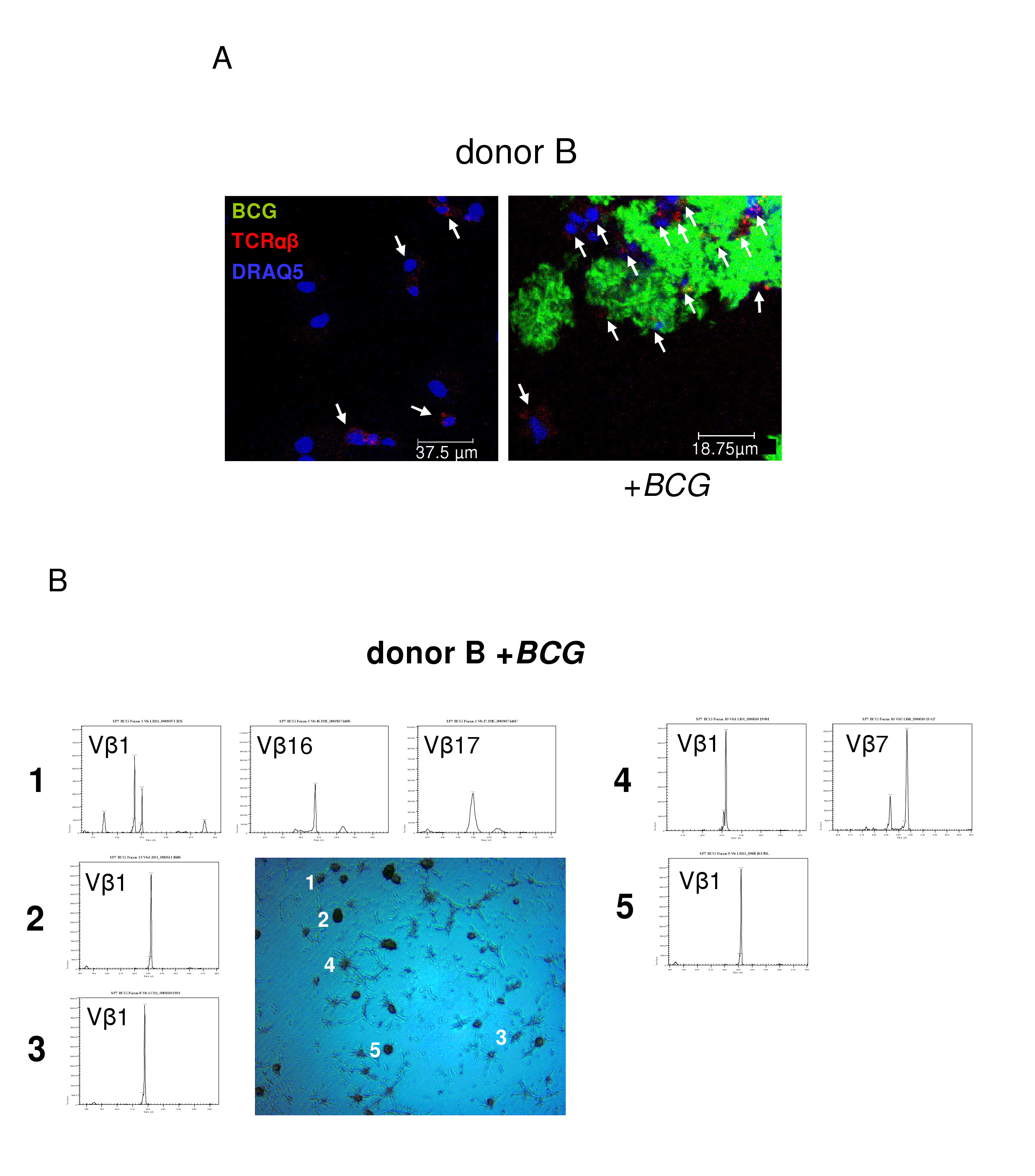

Supplement: Figure S4 — Infection of macrophages with M. bovis BCG induces TCRαβ expression in vitro . (A) Confocal image of a TCRαβ expressing macrophage cluster induced by infection with BCG. Uninfected IFNγ macrophages from the same donor (donor B) are shown left. IFNγ macrophages were incubated in the presence or absence of FITC-labeled BCG for 6 days. White arrows highlight TCRαβ+ macrophages. (B) TCR Vβ repertoire analysis of randomly selected BCG/macrophage clusters from donor B reveals expression of highly restricted TCR Vβ chain repertoires. BCG/macrophage clusters 1-5 were subjected to RT-PCR and CDR3 spectratyping. The identified TCR Vβ repertoires are shown for each individual cluster. Note that next to the Vβ1 only few additional Vβ chains are expressed. The single peaks are indicative of monoclonality. (TIF) [file ppat.1002375.s004.tif]

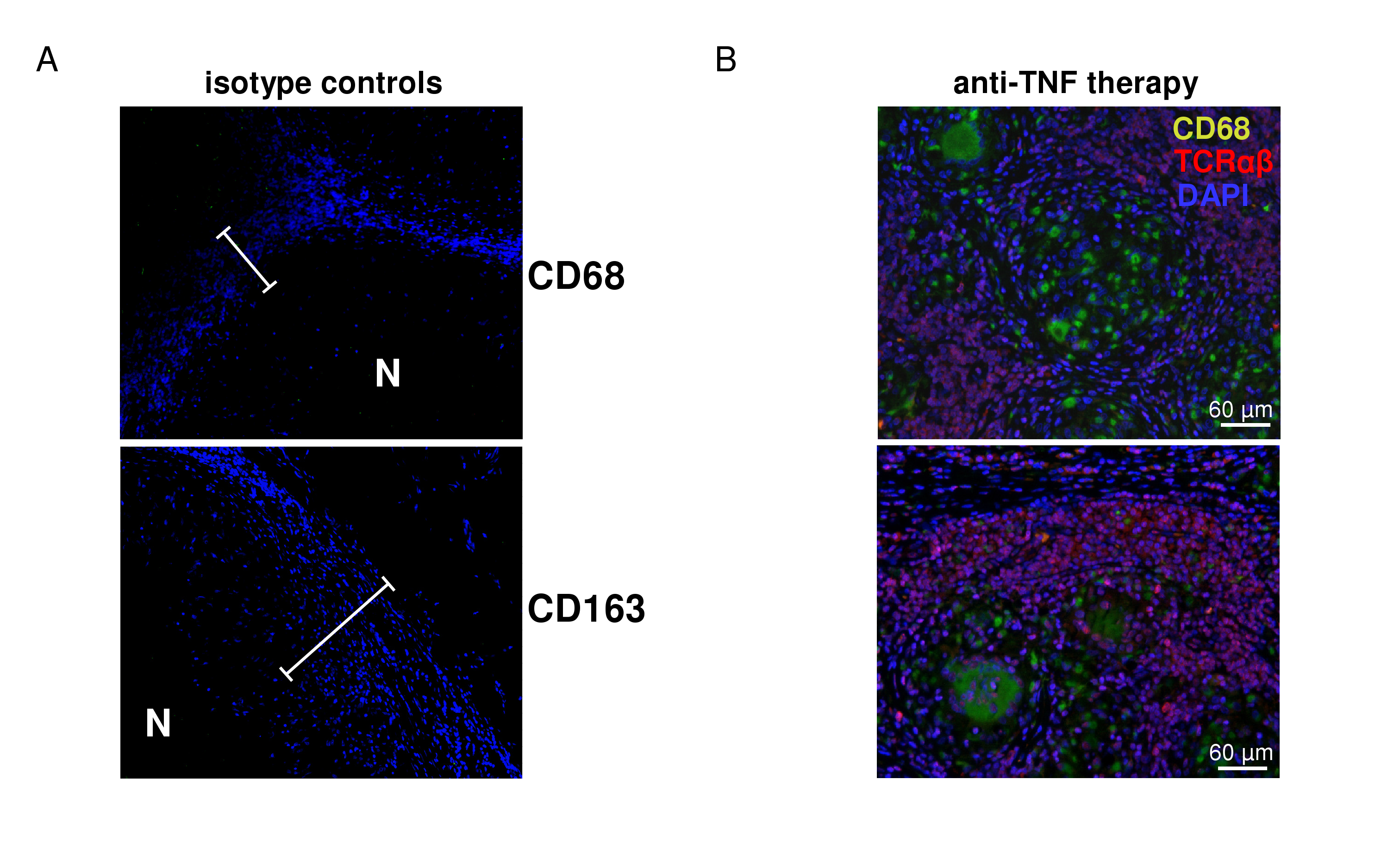

Supplement: Figure S5 — Suppression of macrophage-TCRαβ expression in the tuberculous granulomas of a patient receiving anti-TNF therapy. (A) Isotype control staining of the macrophage markers CD68 and CD163 was performed using the same staining conditions as in Figure 6B. Bars in both images span the inner hostpathogen contact zone. N, necrotic caseous core. 40x. (B) Immunofluorescence microscopy of two tuberculous granulomas present in the lymph node of a patient with therapeutic anti-TNF treatment (adalimumab). Paraffin sections of the granulomas were double-stained for TCRαβ Alexa 555, red) and the macrophage marker CD68 (FITC, green). Nuclei are DAPI-stained (blue). Shown are merged images. Scale bars are indicated. Note the consistent absence of TCR bearing macrophages (TCRαβ+/CD68+, yellow) within the granulomas. The AFB (Acid.Fast Bacilli) test was used to confirm active infection with acid fast mycobacteria in the patient. (TIF) [file ppat.1002375.s005.tif]

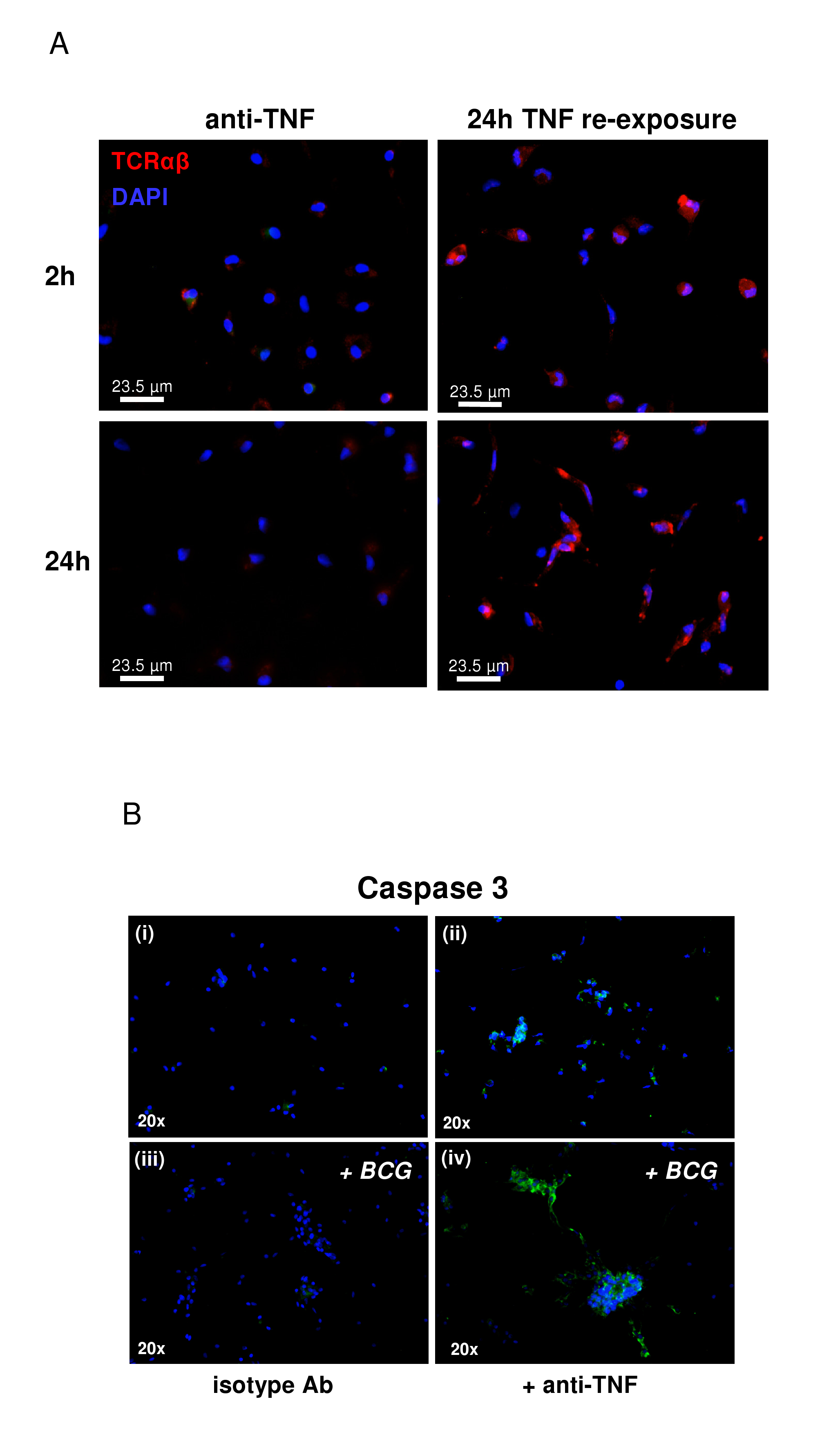

Supplement: Figure S6 — TNF blockade inhibits expression of the macrophage-TCRαβ. (A) Re-exposure to TNF reverses macrophage-TCR suppression induced by TNF blockade. IFNγ activated macrophages were co-cultured in the presence of M. bovis BCG for 24 h followed by incubation with the monospecific anti-TNF antibody infliximab (50 µg/ml) for 2 h and 24 h, respectively. Anti-TNF treatment potently inhibits macrophage-TCR expression (red, Alexa-555 labeled) already after 2 h (left panel). TNFstimulation 10 ng/ml) of anti-TNF treated macrophagesfor 24 h induces TCR expression (right panel). The results shown are representative of two independent experiments. (B) Immunofluorescence staining demonstrates the induction of cleaved caspase 3 (green) in uninfected and M. bovis BCG infected macrophages by TNF blockade. Human CD14+. (TIF) [file ppat.1002375.s006.tif]

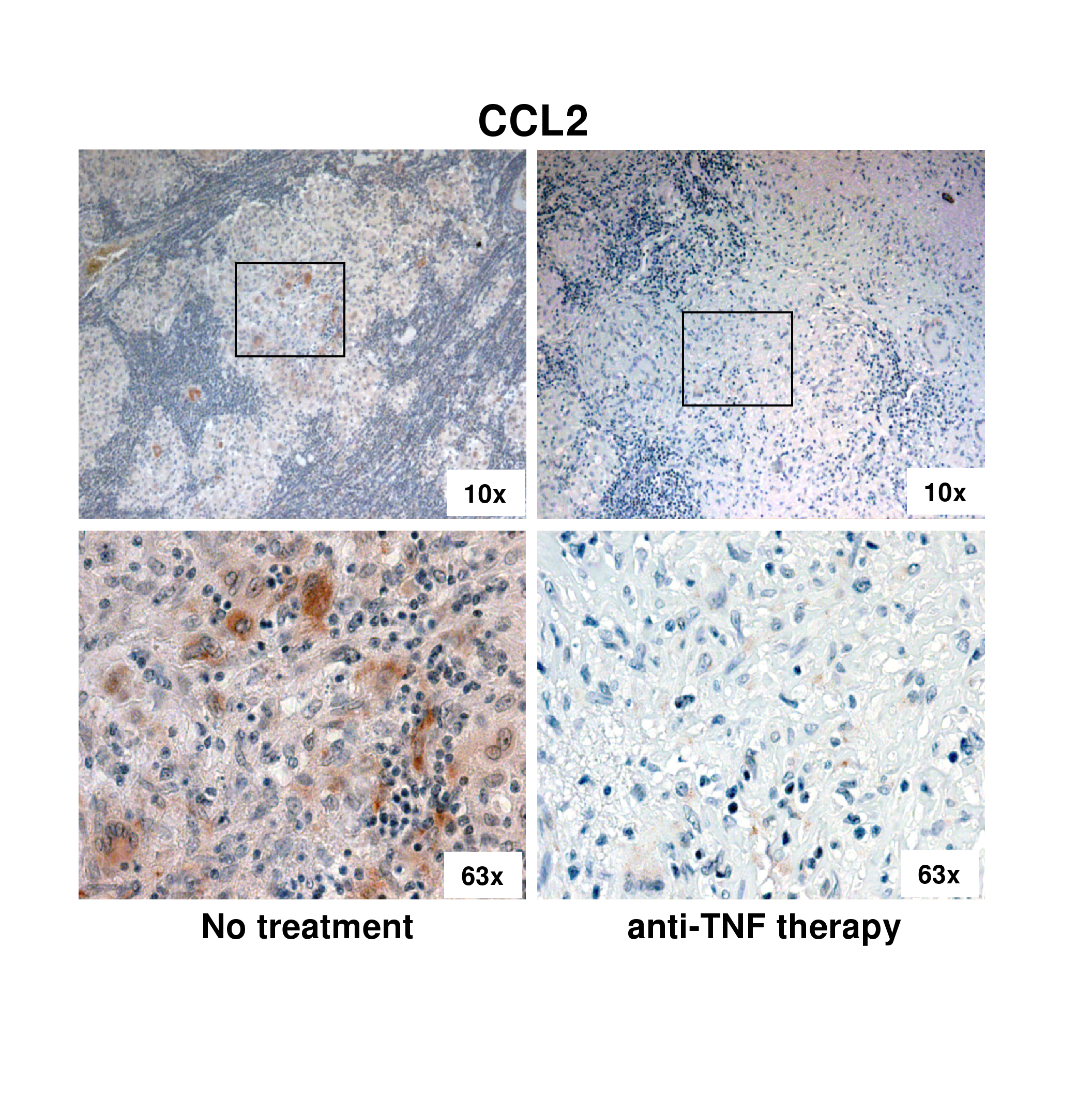

Supplement: Figure S7 — Suppression of macrophage CCL2 expression in the tuberculous granulomas of a patient receiving anti-TNF therapy. Light microscopic immunostaining reveals near absence of CCL2 (DAB, brown) from the lymph node of a patient with therapeutic anti-TNF treatment (adalimumab) (right). A lymph node from an untreated patient displaying intense CCL2 staining is shown as reference (left). Top panel, 10x; the highlighted areas are shown at 63x magnification (bottom). (TIF) [file ppat.1002375.s007.tif]
