# Supplementary material for: A TNF-Regulated Recombinatorial Macrophage Immune Receptor Implicated in Granuloma Formation in Tuberculosis
Source: PLoS Pathog. 2011 Nov 17;7(11):e1002375. doi: 10.1371/journal.ppat.1002375 (PMC3219713; doi:10.1371/journal.ppat.1002375)
Supplement: Table S1 — Synopsis of cytokine/chemokine/growth factor release by IFNγ macrophages. (PDF) [file ppat.1002375.s008.pdf]

Table S1

Synopsis of cytokine/chemokine/growth factor release by IFNγ macrophages

**Effector molecule**	**released from MΦ[1]**	**enhanced CD3 dependent release[2]** ***(> 2 fold relative to isotype control)***
basic FGF	-	-
G-CSF	-	-
GM-CSF	+	-
IFNγ	+	-
IL-1β	+	-
IL-2	-	-
IL-4	+	-
IL-5	-	-
IL-6	+	-
IL-7	+	-
IL-8	+	-
CCL2	+	+
CCL7	+	-
PDGF-BB	+	-
TNF	+	-
CCL5[3]	(-)	-

[1] after 6 days of cultivation in the presence of IFNγ
[2] additional 24 h activation with anti-CD3 antibodies
[3] secretory marker for T cells (control)
