# Supplementary material for: A TNF-Regulated Recombinatorial Macrophage Immune Receptor Implicated in Granuloma Formation in Tuberculosis
Source: PLoS Pathog. 2011 Nov 17;7(11):e1002375. doi: 10.1371/journal.ppat.1002375 (PMC3219713; doi:10.1371/journal.ppat.1002375)
Supplement: Table S2 — Histological and immunohistochemical features of granulomas from patients with pulmonary tuberculosis. (PDF) [file ppat.1002375.s009.pdf]

Table S2

	Organized Granuloma	Granuloma Structure	CCL2	$CD68^+$/ $TCR\alpha\beta^+$ Macrophages	Location of $CD68^+$/ $TCR\alpha\beta^+$ cells	Sex & Age
**1**	Y	Organized. Well-circumscribed with caseous necrosis in centre.	-	+	Surrounding caseous necrosis.	Male 59
**2**	Y	Organized. Well-circumscribed with caseous necrosis in centre.	+	+	Surrounding caseous necrosis.	Male 47
**3**	Y	Organized. Well-circumscribed with caseous necrosis in centre.	-	+	Surrounding caseous necrosis.	Female 68
**4**	Y	Early forming granuloma.	+	+	Positive cells are infiltrating the tissue and within the centre of an early granuloma structure.	Male 43
**5**	N	Several areas of necrosis are evident although the granuloma structure has not formed. Large cellular infiltration.	-	+	Positive cells within clusters of macrophages and T cells.	Male 43
**6**	Y	Organized. Well circumscribed with caseous necrosis in centre.	+	+	Surrounding caseous necrosis.	Male 65
**7**	Y	Large necrotic area and cell infiltration.	-	+	Within the necrotic tissue there are some $CD68^+$/ $TCR\alpha\beta^+$ cells.	Female 67
**8**	Y	Organized. Well circumscribed with caseous necrosis in centre.	+	+	Surrounding caseous necrosis	Male 49
**9**	Y	Organized. Well circumscribed with caseous necrosis in centre.	-	+	Very few $CD68^+$/ $TCR\alpha\beta^+$ cells are present. T cells dominant.	Male 40
**10**	N	Granuloma is not well circumscribed with several necrotic foci containing $CD68^+$ cells within the tissue and massive infiltration of $TCR\alpha\beta^+$ T cells.	-	-		Female 73
**11**	N	T cell infiltration.	-	+	Very few $CD68^+$/ $TCR\alpha\beta^+$ cells are present. T cells dominant.	Male 56
**12**	N	Multiple large necrotic sites, not well circumscribed.	-	-		Female 16
**13**	N	Large cellular infiltration of $CD68^+$ cells and $TCR\alpha\beta^+$ T cells. Disorganized necrosis.	-	-		Female 58
